# Cohorts of immature *Pteropus* bats show interannual variation in Hendra virus serology

**DOI:** 10.1111/1365-2656.70213

**Published:** 2026-02-01

**Authors:** Daniel E. Crowley, Caylee A. Falvo, Chris K. Grant, Benny Borremans, Tamika J. Lunn, Manuel Ruiz‐Aravena, Evelyn Benson, Clifton D. McKee, Daniel J. Becker, Devin N. Jones, Trenton Bushmaker, Y. Tina Yu, Michelle Michie, Adrienne S. Dale, Lianying Yan, Spencer L. Sterling, Christopher C. Broder, Laura B. Goodman, Rasa Petraityte‐Burneikiene, Eric D. Laing, Ina L. Smith, Vincent J. Munster, Agnieszka Rynda‐Apple, Alison J. Peel, Raina Plowright

**Affiliations:** ^1^ Department of Public and Ecosystem Health Cornell University Ithaca New York USA; ^2^ Custom Monoclonals International Corp West Sacramento California USA; ^3^ Wildlife Health Ecology Research Organization San Diego California USA; ^4^ Evolutionary Ecology Group, Department of Biology University of Antwerp Antwerp Belgium; ^5^ Odum School of Ecology University of Georgia Athens Georgia USA; ^6^ Center for the Ecology of Infectious Diseases University of Georgia Athens Georgia USA; ^7^ Department of Wildlife, Fisheries, and Aquaculture Mississippi State University Starkville Mississippi USA; ^8^ Department of Microbiology & Cell Biology Montana State University Bozeman Montana USA; ^9^ Department of Epidemiology, Johns Hopkins Bloomberg School of Public Health Baltimore Maryland USA; ^10^ Department of Biology University of Oklahoma Norman Oklahoma USA; ^11^ Laboratory of Virology, Division of Intramural Research, National Institute of Allergy and Infectious Diseases, National Institute of Health Hamilton Montana USA; ^12^ CSIRO Health and Biosecurity Black Mountain Laboratories Canberra Australian Capital Territory Australia; ^13^ Texas Department of Biological Sciences Texas Tech University Lubbock Texas USA; ^14^ Henry M. Jackson Foundation for the Advancement of Military Medicine Bethesda Maryland USA; ^15^ Department of Microbiology and Immunology Uniformed Services University Bethesda Maryland USA; ^16^ Baker Institute for Animal Health Cornell University Ithaca New York USA; ^17^ Institute of Biotechnology, Life Sciences Center Vilnius University Vilnius Lithuania; ^18^ Centre for Planetary Health and Food Security Griffith University Nathan Queensland Australia; ^19^ Sydney School of Veterinary Science University of Sydney Camperdown New South Wales Australia

**Keywords:** bats, disease ecology, ecoimmunology, serology

## Abstract

1. Understanding the drivers of seasonal disease outbreaks remains a fundamental challenge in disease ecology. Periodic outbreaks can be driven by several seasonally varying factors, including pulses of susceptible individuals through births, changes in host behaviour and social aggregation and variation in host immunity. However, when these potential drivers overlap temporally, isolating their relative contributions to outbreak patterns becomes challenging.
2. We studied Hendra virus, a zoonotic pathogen with seasonal spillovers from bats to horses and humans. Multiple seasonal factors have been hypothesized to drive Hendra virus transmission, including food shortages, birth pulses and changes in host aggregation, but their temporal overlap has made identifying primary drivers difficult.
3. We conducted a 4‐year longitudinal study of *Pteropus* bats to test whether seasonal birth pulses and the resulting influx of susceptible juveniles drive Hendra virus transmission. Using a Bayesian ageing model, we aged sexually immature bats and placed them into birth cohorts. We used our age predictions to model how viral shedding and antibody responses changed as bats aged. We tracked *Bartonella* spp. Infection—a bacterial pathogen requiring close contact for transmission—as an indicator of transmission opportunities within each cohort for comparison.
4. We found no evidence that seasonal birth pulses of immunologically naïve juveniles drove Hendra virus transmission. Two out of three cohorts showed substantially reduced maternal antibody transfer compared to the 2018 cohort, with seroprevalence near zero at our earliest sampling timepoints and showed no clear evidence of synchronized seroconversion. Furthermore, *Bartonella* infection rates were consistent across cohorts, indicating that opportunities for pathogen transmission remained consistent across cohorts despite varying viral shedding patterns.
5. Our findings demonstrate that birth pulses alone cannot explain observed patterns of Hendra virus outbreaks. These results highlight the importance of using multiple lines of evidence to evaluate competing mechanisms underlying seasonal disease dynamics, particularly when potential drivers coincide temporally.

## Graphical abstract

*Pteropus* bat with offspring, photo taken by Manuel Ruiz‐Aravena.

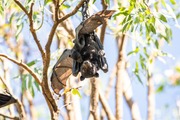

## 1 INTRODUCTION

Pathogens frequently have distinct seasonal outbreaks and transmission patterns. These seasonal outbreaks are caused by specific drivers with distinct mechanisms. For example, annual changes in host social behaviour and aggregation can influence contact rates and transmission opportunities for directly transmitted pathogens (Fine & Clarkson, 1982; Hamede et al., 2009; Silk et al., 2017). Seasonal pulses of births can expand the pool of susceptible hosts, fueling epidemics and facilitating persistence (Altizer et al., 2004; George et al., 2011; Peel et al., 2014; Reijniers et al., 2020). Additionally, seasonal variation in host immunity—driven by resource limitation, reproductive investment or waning immune memory—can impact both transmission probabilities and the duration of infection (Dopico et al., 2015; White et al., 2005; Whiting et al., 2020). However, when multiple seasonal processes coincide temporally, it can be challenging to identify which mechanisms are driving the observed patterns in a pathogen of interest (Lowen et al., 2007, 2008). To address this challenge, we need to empirically test the effects of different seasonal processes.

Progress in understanding how multiple seasonal drivers interact to shape disease transmission patterns has been limited by the scarcity of long‐term ecological data that capture both host and pathogen shedding patterns (Altizer et al., 2006). Insights about seasonal disease patterns have emerged from human systems where long‐term surveillance data enable researchers to identify drivers of disease dynamics (Fine & Clarkson, 1982; London & Yorke, 1973; Pascual et al., 2002). Similar longitudinal studies in wildlife disease systems are rare (Becker et al., 2019; Plowright et al., 2019). This lack of longitudinal studies is concerning because wildlife hosts are significant sources of emerging zoonotic pathogens. Without long‐term studies to understand what drives pathogen transmission in wildlife populations, our ability to predict and prevent pathogen spillover remains limited.

The Hendra virus (HeV) system offers a valuable opportunity to study how multiple seasonal drivers interact to influence pathogen circulation and spillover risk. Hendra virus (HeV) is a zoonotic paramyxovirus that is fatal in horses and humans. *Pteropus* bats in Australia serve as the natural reservoir hosts. Although Hendra virus is typically detected at low prevalence within bat populations (Field et al., 2011; Lunn et al., 2023), there are discrete seasonal pulses of viral shedding, often during the winter months in sub‐tropical regions (Field et al., 2015; Páez et al., 2017). The seasonal drivers of these winter shedding pulses remain unclear. While evidence suggests winter food shortages can trigger increased viral shedding and spillover, multiple factors coincide during winter months, including changes in bat foraging behaviour and movement and the presence of immunologically naïve juveniles (Becker et al., 2023; Eby et al., 2023).

Juveniles are often the most important demographic group for maintaining or amplifying pathogen transmission in human and wildlife systems, especially when immunity is long‐lived (Altizer et al., 2006; George et al., 2011). In many bat species, this effect is amplified by highly synchronized births, creating a ‘birth pulse’ that introduces a rapid influx of immunologically naïve individuals into the population. Once protective maternal antibodies wane in juveniles, this cohort can influence pathogen transmission patterns (Peel et al., 2014; Reijniers et al., 2020). Examples include the impact of birth pulses on Marburg virus infections in Egyptian fruit bats (Amman et al., 2012; Hayman, 2015), seasonal increases in rabies virus incidence in big brown bats (George et al., 2011), adenovirus shedding patterns in Réunion Island free‐tailed bats (Dietrich et al., 2018) and coronavirus shedding in immature bats (Peel et al., 2025). However, the link between juvenile maternal antibody loss and increased Hendra virus shedding has not yet been investigated.

Given these patterns in other bat‐pathogen systems and the seasonal nature of Hendra virus shedding, we tested two hypotheses about transmission seasonality in Australian *Pteropus* bats. First, we hypothesized that most *Pteropus* bats born with protective maternal anti‐Hendra virus IgG antibodies would lose this protection before their first winter, creating a pool of susceptible individuals. This prediction was based on three observations: (1) high prevalence of anti‐Hendra virus IgG antibodies in *Pteropus* adults (Edson et al., 2019), (2) the synchronized 10‐week birth pulse window in sub‐tropical Australia (Mitchell, 2021) and (3) evidence that maternal serum IgG antibodies wane for ~250 days in *Pteropus* bats (Epstein et al., 2013). Our second hypothesis was that following loss of maternal antibodies, a pulse of Hendra virus transmission would occur among immunologically naïve juvenile bats. We predicted this transmission event would manifest as a synchronized seroconversion within juvenile cohorts, detectable through IgG and IgM antibodies acquired from infection.

To assess our hypotheses, we conducted a longitudinal study of Australian *Pteropus* bats over 4 years. We captured bats from multiple birth cohorts and tracked how anti‐Hendra virus antibody levels changed as juveniles aged. To disentangle different seasonal mechanisms, we developed several complementary approaches: (1) We improved age estimates of juvenile bats using morphological metrics to better track cohorts through time and identify when susceptible individuals were emerging within cohorts. (2) We evaluated cohort‐to‐cohort variation in maternal antibody transfer to understand the consistency of protection provided by mothers' antibodies. (3) We separated the effects of seasonal social behaviour from other drivers by measuring *Bartonella* spp., using the bacterium as an independent indicator of transmission opportunities between bats. *Bartonella* is transmitted via flightless bat flies (Nycteribiidae) (Billeter et al., 2012; Low et al., 2022; Morse et al., 2012), requiring close contact for transmission. This makes *Bartonella* suitable for tracking juvenile transmission dynamics, as pteropodid pups are born uninfected and acquire *Bartonella* infection bat fly colonization during the first year of life (Fagre et al., 2023; McKee et al., 2021). (4) We attempted to distinguish between maternally‐acquired and infection‐induced antibodies by measuring anti‐Hendra virus IgM antibodies. (5) Finally, to evaluate whether observed patterns were specific to Hendra virus antibodies or reflected broader immunological processes, we also examined antibody waning and seroconversion against Menangle virus, another paramyxovirus that circulates in Australian flying foxes (Bowden et al., 2001; Philbey et al., 2008). This multi‐faceted approach allowed us to assess the relative importance of different seasonal mechanisms—birth pulses, social contact patterns and immune variation—in driving viral shedding patterns.

## 2 METHODS

### 2.1 Sampling of wild bats

**FIGURE 1:**
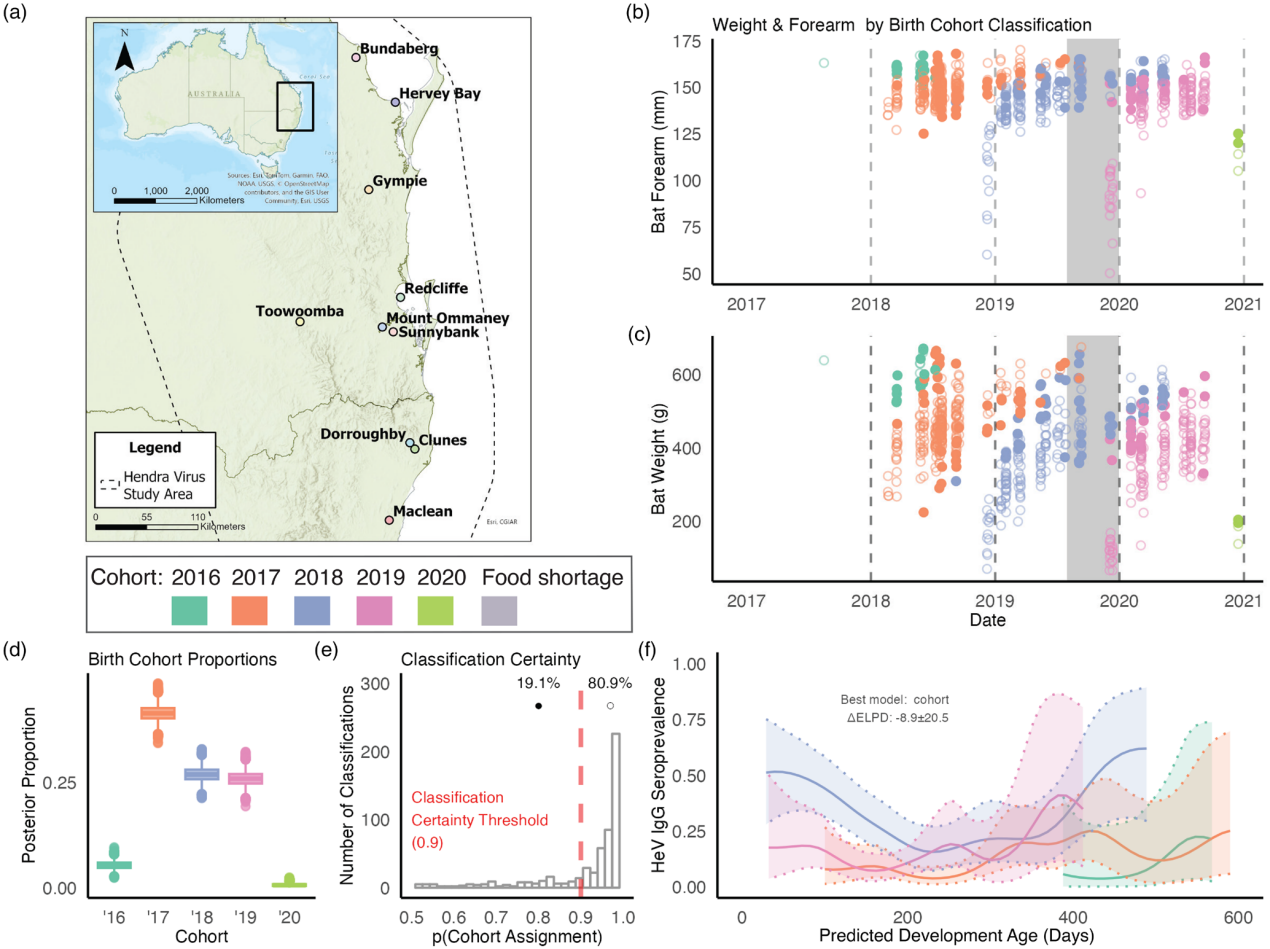
Bayesian cohort classification reveals birth cohort year effects on Hendra virus exposure in juvenile flying foxes (a) Map of sampling locations. Toowoomba and Redcliffe roosts were continuously sampled throughout the study. (b, c) Scatterplots of capture date versus bat forearm (b) and weight (c) from our longitudinal study of subadult bats. Adults are excluded. Each datapoint's colour represents birth cohort assignment by our Bayesian mixture model. Closed circles represent bats with posterior classification probability ≤ 0.9 (uncertain assignments); open circles represent bats with posterior classification probability >0.9 (high certainty assignments). Grey shading indicates the food shortage event period. (d) Birth cohort proportions estimated by the Bayesian mixture model, showing the relative abundance of each cohort in the population. (e) Distribution of posterior classification probabilities from the Bayesian mixture model. The dashed red line at 0.9 indicates the threshold used to distinguish high certainty (open circles) from uncertain (closed circles) classifications in panels (b, c). Percentages show the proportion of classifications above and below this threshold. (f) Juvenile bat cohort patterns of anti‐Hendra virus IgG seroprevalence. The *x*‐axis represents predicted developmental age (days). Estimates are from our Gaussian smoothing model. Shaded regions represent 95% credible intervals. Birth cohort classification (2016, 2017, 2018, 2019 and 2020) is based on the year the bat was born.

We caught wild black flying foxes (BFF, *Pteropus alecto*) and grey‐headed flying foxes (GHFF, *Pteropus poliocephalus*) between August 2017 and December 2020, as described previously (Hansen et al., 2022; Peel et al., 2022). Bats were caught during periods of typical and atypical food availability, including a food shortage event in the fall of 2019 (Eby et al., 2023). Sampling was conducted five times per year at continuously occupied roost sites in Toowoomba and Redcliffe, Queensland, Australia. In addition to these two sites, we conducted infrequent sampling of roost sites occupied by nomadic influxes, including Gympie (summer 2019), Mount Ommaney (summer 2019), Maclean (winter 2018) and Hervey Bay (winter 2018, winter 2020), mapped in Figure 1a. Catching sessions lasted from 3 to 4 days and targeted a total of 60 bats each. All animal handling was conducted under approval from the Griffith University Animal Ethics Committee (Certificate: ENV/10/16/AEC and ENV/07/20/AEC) and the Montana State University IACUC (#201750). Personal protective equipment and biosafety protocols followed best‐practice guidelines (e.g. IUCN Bat Specialist Group, 2021; Wildlife Health Australia, 2020).

In the field, we collected morphological metrics from each bat, including but not limited to forearm length (millimetres) and weight (grams). Bats were also assigned an age classification of ‘dependent pup’, ‘juvenile’, ‘subadult’ and ‘adult’ based on a visual assessment of size and sexual maturity in conjunction with forearm and weight measurements (Pietromonaco et al., 2025). Dependent pups were suckling individuals that were captured attached to their mothers. Juveniles were captured independently from their mothers and were visually assessed as non‐reproductive (the genitals had not undergone sexual development), with sharp teeth with little discoloration and were smaller than full body size, based on weight and forearm measurements. To classify bats as sub‐adults, we visually assessed if a bat was still non‐reproductive, but was at or near full body size, based on weight and forearm measurements. To classify bats as adults, we visually assessed if bats were reproductive (obvious development of genitals) and were full body size, based on weight and forearm measurements. Adults often also had stained and worn teeth. We captured 674 sexually immature bats, including 34 bats classified as ‘dependent pups’, 414 bats classified as ‘juveniles’ and 229 bats classified as ‘sub‐adults’. Not all bats were eligible for every analysis; exclusion criteria are listed in Figure S1.

### 2.2 Classifying non‐adult bats into respective cohorts using morphological data

Within our study area, *P*. *alecto* and *P. poliocephalus* birth pulses occur annually with peaks around mid‐to‐late October (Mitchell, 2021; Vardon & Tidemann, 1998). While small immature bats sampled immediately after this birth pulse can be easily identified as belonging to the most recent cohort, ageing and cohort classification in the field become challenging for larger juveniles and subadults (Pietromonaco et al., 2025).

To address this challenge, we developed a Bayesian mixture model (Figure S2D) that uses body measurements to estimate age and assign bats to specific birth cohorts, named according to the year of birth. This approach allowed us to objectively classify bats while accounting for natural variation in growth rates. The model incorporated four key pieces of information: forearm length (fa), weight (wt), sex and sampling date (y). These variables have been previously used to model *Pteropus* bat growth, though only for single birth cohorts (Todd et al., 2018; Vardon & Tidemann, 1998).

In our model, implemented using Rstan version 2.32.5, each animal *i* could have been born in one of k birth cohorts $\omega_{k}$ with probability $\theta_{k}$(where θ is a simplex vector that sums to 1). The transformation of weight and forearm measurements (to powers of two and six, respectively) was determined through exploratory analysis to achieve a linear correlation and normal distribution of the data (Figure S2B).




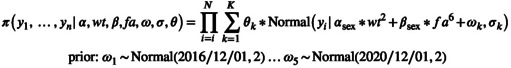




Prior distributions for the model reflected known *Pteropus* bat biology. Birth pulse timing priors ( *ω* ) were informed by documented breeding seasonality, and the prior on cohort assignment probability (*θ*) was set as Dirichlet(1,5,5,5,1), reflecting expected proportions from each cohort based on our sampling timeline (~30% chance from 2017 to 2019 cohorts, ~5% chance from 2016 or 2020 cohorts) (Figure S2C).

The study did not capture the dependent pups from the 2016 and 2017 cohorts. Thus, we only observed older juveniles and subadults from these cohorts. To improve model performance, we simulated data for 20 dependent pups for these cohorts using the mean and standard deviation from observed dependent pups, removing these simulated data after model fitting (Figure S2A). Model convergence was assessed using MCMC trace plots, R‐hat values and examination of joint posterior distributions.

We deliberately avoided capturing pups in October to minimize stress on late‐pregnant females and newborns. Consequently, our sampling of each cohort began in early December, approximately 6–8 weeks after the peak birthing period (Figure S2A). As we could not observe bats from birth, our model estimates a ‘predicted developmental age’ ($\phi_{i}$) rather than absolute age. $\phi_{i}$ is a standardized developmental metric measuring days of growth since our first opportunity to sample that cohort (early December). To estimate $\phi_{i}$ using this formula with parameters from the model's posterior estimates: $\phi_{i}=\alpha_{\mathrm{sex}}*\text{weigh}t_{i}+\beta_{\mathrm{sex}}*\text{forear}m_{i}$.

Given that our study included two *Pteropus* species (*P. alecto* and *P. poliocephalus*), we assessed whether combining species in our analyses was justified. We compared model performance using data restricted to black flying foxes (*P. alecto*) only, the majority of our samples, to a model using data restricted to grey‐headed flying foxes (*P. poliocephalus*). Growth model parameters were compared between species (Figure S3).

### 2.3 Validation of cross‐reactive anti‐feline IgM antibodies for use with Pteropus serum

There are no commercially available anti‐*Pteropus* IgM mAbs, so we first needed to identify and validate an anti‐IgM mAb for *Pteropus* bats. We screened commercially available anti‐feline IgM and IgG mAbs for cross‐reactivity with *P. alecto* serum antibodies. All screened mAbs were developed by Custom Monoclonals International and have been previously described (Grant, 1995). Sera for IgM validation came from *P. alecto* bats sourced from the Lubee Bat Facility (Florida, USA) or the Australia Zoo Wildlife Hospital (QLD, Australia). Imported sera from wild bats in Australia were inactivated with 2 or 8 megarads of gamma irradiation according to standard procedures (Feldmann et al., 2019). IgM reagents were assessed using a combination of affinity and size exclusion chromatography columns in conjunction with ELISA (Figure S4A) and Western blot (Figure S4B). For more details see Supporting Information: Methods Section 1.

### 2.4 Serology: IgG And IgM measurements and cutoffs

To investigate Hendra virus transmission waning and seroconversion in these age‐classified cohorts, we conducted detailed serological analyses. Blood for IgG measurements was collected from all bats except for dependent pups, as described previously (Hansen et al., 2022). Serum was allowed to clot for at least 4 h at ambient temperature before being spun down, aliquoted, and then stored at −80°C until testing. Prior to testing, serum samples were thermally inactivated at 56°C for 30 min and stored at −80°C. For antibody measurements, serum samples were diluted 1:500 in PBS and added to a multiplex of antigen‐coupled magnetic microspheres. The antigen‐beads were washed three times with PBS‐Tween 20 between each incubation for 30 min.

Detection of IgG antibodies was conducted using a 1:1 mixture of biotinylated‐protein A and biotinylated‐protein G (2 μg/mL, Thermofisher). Antigen–antibody complexes were further incubated with streptavidin‐phycoerythrin (1 μg/mL, Thermofisher), and analysed using a MagPix multiplexing machine (Luminex).

For IgM, we processed serum samples the same as IgG, except we used the CM7:Biotin (1.7 μg/mL) instead of protein A/G. Again, antigen–antibody complexes were further incubated with streptavidin‐phycoerythrin (1 μg/mL, Thermofisher) and analysed using a MagPix multiplexing machine (Luminex).

We measured the Hendra virus‐g1 variant attachment glycoprotein (G) as an antigen. The expression and purification of the Hendra virus G antigen as soluble and secreted oligomeric ectodomains in their native‐like tetrameric conformation and procedures for coupling to magnetic microspheres (MagPlex, BioRad) for multiplex serology have been previously described (Cheliout Da Silva et al., 2021; Yan et al., 2023). Anti‐Hendra virus‐g1 and ‐g2 antibodies would be expected to be cross‐reactive with Hendra virus‐g1 G protein, meaning that serological results are considered to encompass both genotypes. Several monoclonal antibodies (mAbs) to both the G and F glycoproteins have been shown to be cross‐reactive (Annand et al., 2022; Wang et al., 2022).

To evaluate whether our observed patterns were specific to Hendra virus or reflected broader immunological processes, we also examined antibody responses over time against Menangle virus, another paramyxovirus that circulates in Australian flying foxes. Menangle virus provides a useful comparison as it shares similar host species, is also transmitted through urine and is a known zoonosis (Bowden et al., 2001; Philbey et al., 2008).

Both the Menangle virus and Hendra virus antigen measurements were part of a multiplexed serology platform that included additional viral antigens. These antigens were produced in human FreeStyle 293‐F cells (Thermo Fisher Scientific, Waltham MA) by stable cell‐line generation (henipaviruses, filoviruses and, pararubulaviruses) or in Expi293F cells (Thermo Fisher Scientific) by plasmid transfection (coronaviruses). In addition to Hendra virus, the expression and purification of Nipah virus (NiV), Cedar virus (CedV), Ghana virus (GhV) and Mòjiāng virus (MojV) envelope attachment glycoprotein (G) antigens as soluble and secreted oligomeric ectodomains in their native‐like tetrameric conformation and procedures for coupling to magnetic microspheres (MagPlex, BioRad) for multiplex serology have been previously described (Cheliout Da Silva et al., 2021; Yan et al., 2023). Likewise, the attachment hemagglutinin‐neuraminidase (HN) glycoproteins of pararubulaviruses (Grove virus (GroV), Yeppoon virus (YepV) and Menangle virus (MenV)) were similarly designed and produced as soluble tetrameric ectodomains. The expression and purification of the envelope glycoproteins of filoviruses (Zaire ebolavirus (EBOV), Bundibugyo ebolavirus (BDBV), Bombali ebolavirus (BOMV), Marburg marburgvirus (MARV) and Ravn virus (RAVV)) as soluble trimeric fusion glycoprotein (GP) ectodomains, and the spike (S) glycoprotein trimers of coronaviruses severe acute respiratory syndrome (SARS) and Middle East respiratory syndrome (MERS) coronaviruses (CoV) (SARS‐CoV, SARS‐CoV‐2 and MERS‐CoV), and their application in multiplex serology have been previously described (Clifton et al., 2021; Laing et al., 2018; Pulscher et al., 2022). Unlike the previous antigens, the expression and purification of Menangle virus N antigen was previously achieved in yeast *Saccharomyces cerevisiae*, generating nucleocapsid‐like particles as previously described (Juozapaitis et al., 2007). While we measured antibodies to these viral antigens, we focused temporal analyses only on Hendra virus (our primary pathogen of interest) and Menangle virus (as a comparison paramyxovirus to test whether patterns were Hendra‐specific). The remaining antigens served to assess antibody cross‐reactivity and validate the specificity of our serological measurements (Figure S5).

We tested three approaches to classify antibody levels as positive or negative: a two‐part gamma distribution, a two‐part normal distribution and a three‐part normal distribution. The three‐part model tested whether we needed an ‘intermediate’ category. Models were compared using expected log pointwise predictive density leave‐one‐out cross‐validation (ELPD‐LOO‐CV) implemented in the LOO package (version 2.8.0). Models were adapted from previously published methods (Brook et al., 2019; Edson et al., 2019; Peel et al., 2013). For IgG and IgM, we transformed the antibody readout (reported as a median fluorescence intensity [MFI]) values. We first took the natural log of MFI (lnMFI) and then, to better fit the gamma distribution, we set the minimum lnMFI value to zero for each antigen (Figures S6A and S7A). Mixture model fit was assessed using the trace plots and joint posteriors from MCMC sampling and ELPD‐LOO‐CV. Cutoffs for seropositivity were derived from the best model fit for each antibody type. For IgG measurements, we used a conservative cutoff of 3 standard deviations above the mean of the seronegative distribution to minimize false‐positives, adapted from past work (Edson et al., 2019; Peel et al., 2013). To classify IgM MFI as IgM seropositive, we used a >50% probability of belonging to the mixture distribution with the higher MFI (Figure S7A). This IgM cutoff is less conservative than the IgG cutoff, but we felt this was appropriate because of the lower affinity of mammalian IgM antibodies.

To address potential IgM cross‐reactivity concerns in our Luminex platform, we ran a principal components analysis (PCA). PCA provides a breakdown of the correlation structure of high‐dimensional datasets, enabling us to assess how lnMFI levels for all antigens in our data set are correlated. For the PCA, all lnMFI values were rescaled to have a mean of 0 and standard deviation of 1. Principal components (PCs) were assessed by using an eigenvalue eigenvector decomposition of the lnMFI covariance plot. Individuals that were missing measurements for specific antigens were not included in the PCA.

### 2.5 Hendra virus PCR detection

We tested for the presence of Hendra viral RNA in the urine of bats. Urine was collected from captured bats and placed into AVL buffer (Qiagen) or viral transport media (VTM). Target volumes were 140 μL of urine into 560 μL AVL and 200–1000 μL urine into 1000 μL VTM. Samples were transported via Cryoshipper charged with liquid nitrogen or portable −80°C shipper and then stored at −80°C.

Viral RNA was extracted from individual urine samples and screened for Hendra virus genotype‐1 (Hendra virus‐g1) and Hendra virus genotype‐2 (Hendra virus‐g2), using a qRT‐PCR assay as previously described (Lunn et al., 2023; Peel et al., 2022). Briefly, the QIAamp Viral RNA kit and QIAcube HT system (Qiagen) were used to extract RNA. RNA was eluted into 150 μL of TE buffer. Hendra virus genotype‐1 (HeV‐g1) duplex qRT‐PCR assay was used for the detection of viral RNA. The qRT‐PCR used 10 μL RNA, 900 nM and 250 nM final concentrations of primer and probe, respectively, and 10 μL of TaqMan Fast Virus 1‐Step master mix with a QuantStudio 6 flex Real‐Time PCR instrument (Applied Biosystems). Any Hendra virus qRT‐PCR Ct value less than or equal to 40 was classified as positive. Due to the low number of Hendra virus‐g2 detections (Peel et al., 2022), only Hendra virus‐g1 was used in the analyses.

In addition to the presence of viral RNA, we tested if there was an association between being Hendra virus PCR positive and having antibodies against Hendra virus using a logistic regression model. In this model, cohort assignment and sampling location were included as grouping variables. All logistic regression models were run using Rstan version 2.32.5. Sample sizes are included in Figure S1.

### 2.6 Bartonella detection

To provide context for our Hendra virus findings and validate transmission opportunities across cohorts, we examined infection patterns of *Bartonella* spp. Transmission of *Bartonella* in *P. alecto* is hypothesized to be through bat flies (Nycteribiidae), the predominant ectoparasites found on similar hosts (Fagre et al., 2023; McKee et al., 2024). The likely vector is a *Cyclopodia* species based on previous surveys of bat flies on *P. alecto* (Vidgen et al., 2017). Nycteribiids in general and *Cyclopodia* spp. in particular are wingless (Dittmar et al., 2015; Mehlhorn & Klimpel, 2014) and are only capable of moving between hosts by crawling between bats within a roost. Therefore, horizontal transmission of *Bartonella* requires close contact between bats, similar to the presumed transmission route for Hendra virus, though neither has been experimentally confirmed.

As our study focused on juvenile bats during their first year of life, interpretation issues resulting from chronic *Bartonella* infections should be minimal. Pups are born uninfected and become colonized by nycteribiid bat flies within their first year, leading to increasing *Bartonella* prevalence over time (Fagre et al., 2023; McKee et al., 2021, 2024). This age‐specific infection pattern makes *Bartonella* particularly suitable for tracking transmission opportunities in juvenile cohorts.

To detect the presence of *Bartonella*, two to three drops of whole blood from bats were spread directly onto Whatman FTA cards, which were stored at ambient temperature until DNA extraction. Nested PCR for *Bartonella* spp. was performed as previously described (Birtles & Raoult, 1996; Norman et al., 1995). We modelled the prevalence of *Bartonella* spp. PCR positive bats as juvenile bats aged using the same methods we employed for our IgG serology data. To confirm the identity of circulating *Bartonella* spp., we sequenced the *Bartonella* amplicons. For details on sequencing, see Supporting Information: Methods Section 2.

### 2.7 Temporal patterns of serological and pathogen measurements

We used our predicted developmental age to understand how our pathogen and antibody measurements changed as sexually immature bats grew, and how these transmission and seroconversion events differed across cohorts. First, we compared the anti‐Hendra virus IgG temporal seroprevalence patterns between the 2016, 2017, 2018 and 2019 birth cohorts. The 2020 cohort was omitted because it contained only dependent pups, from which we did not draw blood. We modelled infection prevalence and antibody seroprevalence using a Bayesian Gaussian smoothing process in Rstan version 2.32.5. The full model is written out in Figure S8A and draws from the model's posterior distributions in Supp. Figure S8B. To assess intercohort variation in the seroprevalence over time, we compared models that did and did not include cohort assignments. For these model comparisons, we used ELPD‐LOO‐CV. Models were only compared when the Pareto *k* values for individual observations were below 0.7.

## 3 RESULTS

### 3.1 Cohort model classifies bats into five cohorts

The Bayesian mixture model successfully classified bats into five birth cohorts (Figure 1b,c). The model showed strong classification confidence, with 72% of bats assigned to a cohort with a posterior probability >90% (Figure 1d,e). The remaining 28% of bats had posterior probabilities between 50% and90%, with no bat having less than a 50% probability of cohort assignment (Figure 1b,c).

To validate our approach of combining both *Pteropus* species in our analyses, we compared model outputs using *P. alecto* or *P. poliocephalus* only. The growth model coefficients showed substantial overlap between the single‐species analyses, with similar parameter estimates for both weight and forearm measurements across sexes (Figure S3). Based on these findings, which demonstrated minimal species‐specific differences, we used a combined‐species approach.

### 3.2 Technical validation of IgG serological assay

A two‐part gamma mixture model best classified the anti‐Hendra virus‐G IgG MFI data (Figure S6D), providing a seropositivity cutoff value of 2.17 (Figure S6A). Model diagnostics indicated satisfactory convergence, with MCMC trace plots (Figure S6C) and joint posterior distributions supporting model fit (Figure S6E). The adult anti‐Hendra virus IgG seropositive percentage in our dataset, estimated using our lnMFI cutoff, ranged from 50% to 70% over the course of the study, which was in line with previously reported rates in Australian *Pteropus* bats (Boardman et al., 2020; Breed et al., 2011; Edson et al., 2019; Plowright et al., 2008).

To validate our approach of combining both *Pteropus* species in our analyses, we compared model outputs using *P. poliocephalus only* versus *P. alecto only*. The mixture model analysis for anti‐Hendra virus IgG yielded comparable seropositivity cutoffs (*P. poliocephalus only*: 2.54 lnMFI; *P. alecto* only: 2.34 lnMFI) (Figure S3). Based on these findings, which demonstrated minimal species‐specific differences, we continued with a pooled species approach.

### 3.3 High intercohort variation in anti‐Hendra virus IgG temporal seroprevalence

We found statistical support for intercohort variation in anti‐Hendra virus IgG seroprevalence (ELPD‐LOO‐CV estimate ± SE with cohorts: −228.5 ± 14.4; without cohorts: −237.4 ± 14.4; model differences: −8.9 ± 20.0). When we looked at the individual cohorts, only the 2018 cohort exhibited temporal seroprevalence patterns that supported our original hypothesis (i.e. loss of maternal antibodies followed by a population‐wide seroconversion period; Figure 1f; raw data shown in Figure S9). Further model validation results are presented in Supporting Information: Results Section 2.

After observing this intercohort variation in anti‐Hendra virus IgG temporal seroprevalence patterns, we developed a series of a posteriori hypotheses (Table 1). These hypotheses were developed to help us interpret our IgG finding and rule out potential explanations of these data.

**TABLE 1:** A posteriori hypotheses.

	A posteriori hypothesis	Prediction and rationale	Outcome
1	Intercohort variation in Hendra virus serological dynamics was the result of intercohort variation in opportunities for horizontal pathogen transmission	There will be similar intercohort variation in other pathogens, such as *Bartonella* spp. In this analysis, we assumed that *Bartonella* spp. transmission also requires close contact While *Bartonella* can cause chronic infections, pups are born uninfected (McKee et al., 2021), and our focus on juveniles during their first year minimizes confounding from chronic infections. Additional, *Bartonella* spp. is spread through flightless nycteribiid bat flies that can only move between hosts by crawling, thus requiring close contact between bats (Dittmar et al., 2015; Fagre et al., 2023; Hou et al., 2018; McKee et al., 2024; Mehlhorn & Klimpel, 2014; Morse et al., 2012)	Based on *Bartonella* results (Figure 2) we rejected this hypothesis. There was no evidence for intercohort variation in *Bartonella* detection, our proxy measure for the conditions for horizontal pathogen transmission
2	Our sampling events were too infrequent to capture the serological dynamics we expected (i.e. loss of maternal antibodies followed by a population wide seroconversion event)	Temporal variation in IgG seroprevalence for another antigen, Menangle virus, will show similar intercohort variation	Based on our Menangle virus IgG patterns, we could not reject this hypothesis completely. There was intercohort variation in Menangle virus IgG, albeit the evidence was weaker than for anti‐Hendra virus IgG and dependent on the antigen (N vs. HN)
3	Our anti‐Hendra virus IgG measurements were not a proxy for Hendra virus transmission events in the population	Anti‐Hendra virus IgG will not correlate with Hendra virus RNA detection or anti‐Hendra virus IgM antibodies	Based on the correlation between IgG and RNA detection in our logistic regression, we can reject this hypothesis. Patterns in anti‐Hendra virus IgG seroprevalence are reflecting acute Hendra virus infections in juvenile and subadult bats. However, our IgM data was too cross‐ reactive to be applied in this context
4	Juvenile cohorts did not possess maternal antibodies, because pregnant females did not have circulating serum antibodies	There will be variation in IgG levels among pregnant mothers that aligns temporally with the cohorts which lacked evidence for maternal antibodies	We can reject this hypothesis; we observed consistent levels of anti‐Hendra virus IgG antibodies in female adults

### 3.4 No support for significant intercohort variation in opportunities for horizontal transmission

**FIGURE 2:**
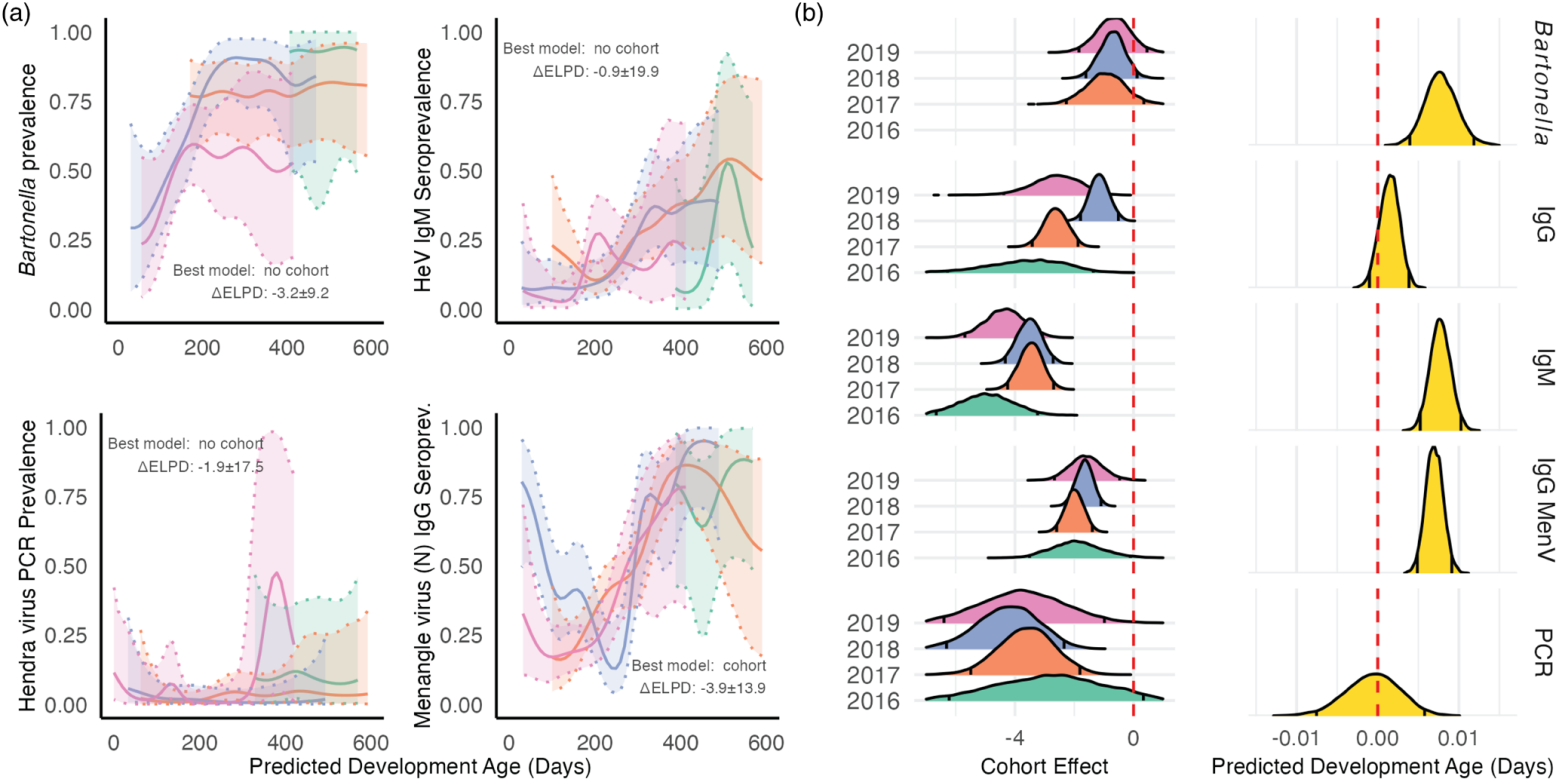
Model output for serological and pathogen detection patterns, (a) including juvenile bat cohort prevalence dynamics of Bartonella spp., anti‐Hendra virus IgM, Hendra virus RNA and anti‐Menangle virus‐N IgG. On the X axis is developmental age since initial cohort sampling. The estimates are from our Gaussian smoothing model. The shaded region around the prevalence estimate represents the 95% credible interval from the Gaussian smoothing model. Cohort classification labels (i.e. 2016, 2017, 2018 and 2019) are based on the year the bat was predicted to be born. (b) Posterior draws from the effect estimates from a logistic regression with the dependent variable: Detection of anti‐Hendra virus IgG, Bartonella spp., anti‐Hendra virus IgM, Hendra virus RNA and anti‐Menangle virus‐N IgG. The posterior distributions shown are the log odds for predicted developmental age (days) and the predicted odds for each assigned cohort.

We utilized *Bartonella* detection as an independent indicator of conditions required for horizontal pathogen transmission in our study populations (Table 1, a posteriori hypothesis 1). *Bartonella* bacteria are transmitted through close contact via flightless bat flies, making them a proxy for transmission opportunity. We found that most bats within each cohort became infected with *Bartonella* within their first year of life, and importantly, this pattern was consistent across all cohorts (Figure 2a,b). The lack of intercohort variation in *Bartonella* infection rates (ELPD‐LOO‐CV estimate ± SE with cohorts: −127.5 ± 6.5, without cohorts: −124.3 ± 6.5, model difference: −3.2 ± 9.3) suggests that opportunities for horizontal pathogen transmission were similar across cohorts. This consistent increase in *Bartonella* prevalence during the first year of life aligns with previous observations of juvenile bat colonization by bat flies (Fagre et al., 2023; McKee et al., 2024).

We assessed the diversity of *Bartonella* genotypes among our sample, as past work in rodents found that different genotypes of *bartonellae* had different temporal patterns of infections in the same host population (Brook et al., 2017). We found support for two monophyletic clades in our data, which were consistently found through our samples (Supporting Information: Results Section 3 and Figure S10). We identified two monophyletic clades in our data, with clade B consistently more prevalent than clade A (86% vs. 14% on average) across all demographic factors including location, time, age and sex (Supporting Information: Results Section 3, Figure S10, and Table S1). This stable ratio between clades, maintained across all cohorts and sampling conditions, suggests both clades share similar temporal patterns in transmission events, with clade A maintained at lower prevalence in the population.

### 3.5 Limited intercohort temporal variation in Menangle virus seroprevalence

We found a two‐part gamma mixture model best classified the anti‐Menangle virus‐N IgG MFI compared to the two‐part normal and three‐part normal mixture model (Figure S11A,C). The gamma mixture model for anti‐Menangle virus‐N IgG provided a cutoff value of 3.27 lnMFI, based on 3 SD above the mean of the seronegative distribution. The cross‐reactivity and specificity for Menangle virus‐N have not yet been validated. However, as a nucleoprotein, it is expected to be more broadly cross‐reactive than the Menangle virus binding antigen (HN). No mixture model fit the MFI for Menangle virus‐HN MFI better than a single gamma distribution, despite many individuals having high MFI values (Figure S11B,D). To establish a cutoff, we used the second‐best fitting model, the two‐part normal distribution, which provided a cutoff of 2.2 lnMFI.

Within the first year of life, all cohorts developed antibodies towards the anti‐Menangle virus‐N (Figure 2a) and, to a lesser extent, HN proteins (Figure S11E), partially supporting one of our a posteriori hypotheses (Table 1, a posteriori 2). There was no statistical support for including cohort in our model of anti‐HN proteins (ELPD‐LOO‐CV estimate ± SE: with cohort: −204.2 ± 15.5, without cohort: −204.1 ± 15.4, model difference: −0.1 ± 21.9). When we modelled the Menangle virus‐N protein, we found statistical support for including cohort in our model (ELPD‐LOO‐CV estimate ± SE: with cohort: −337.2 ± 10.4, without cohort: −341.4 ± 9.9, model difference: −4.3 ± 13.9), although the statistical support was weaker than for anti‐Hendra virus IgG. Only the 2018 cohort showed a clear loss of maternal anti‐Menangle virus‐N antibodies for both antigens.

### 3.6 Anti‐Hendra virus IgG MFI correlated with Hendra virus RNA detection

**FIGURE 3:**
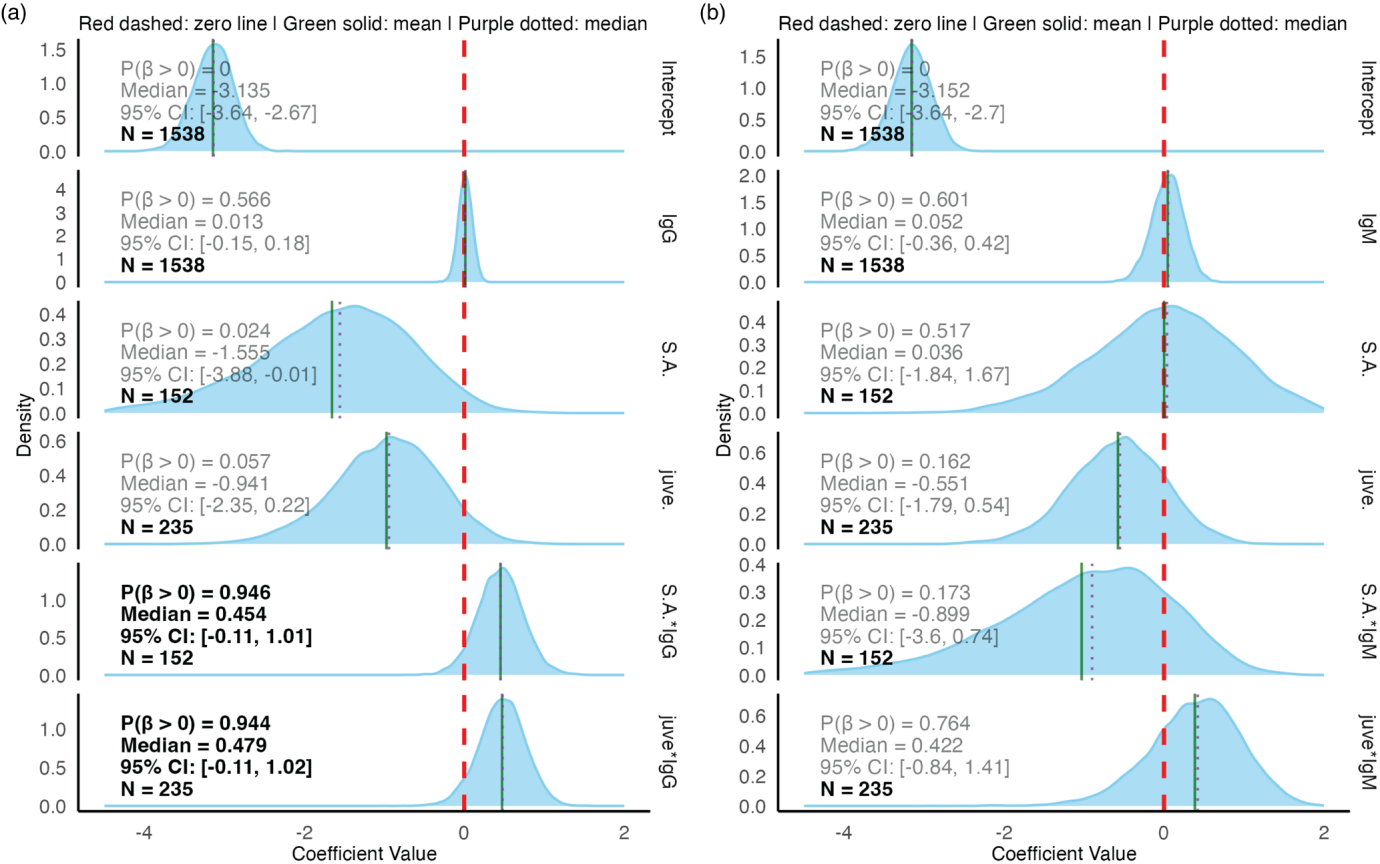
Bayesian logistic regression analysis of Hendra virus RNA detection associations with antibody levels across age classes. Posterior distributions of regression coefficients from logistic models examining the relationship between Hendra virus qRT‐PCR detection (binary outcome) and anti‐Hendra virus IgG (a) or IgM (b) lnMFI values, with age class interactions. Each density plot shows the posterior distribution for individual model parameters: Intercept (baseline log‐odds), main effects for antibody levels (IgG/IgM) and age classes (sub‐adult [S.A.] and juvenile [juve.] relative to adult reference), and interaction terms (Age class*IgM, Age class*IgG). Vertical lines indicate the null effect (red dashed), posterior mean (green solid) and posterior median (purple dotted). Text annotations display the probability of positive association [P(*β* > 0)], posterior median, 95% credible intervals and sample sizes and are highlighted in bold to emphasize covariates when P(*β* > 0) exceeds or nears 0.95%.

We next used Hendra virus RNA detection to validate our anti‐Hendra virus IgG measurements to test our next a posteriori hypothesis (Table 1, a posteriori hypothesis 3) (Figure 3). Among juvenile and subadult bats, the odds of qRT‐PCR detection increased with anti‐Hendra virus IgG lnMFI (PE & CI Juvenile: 0.48 {0.02, 0.88}; Subadult: 0.48 {0.05, 0.90}). This was not the case for IgM lnMFI (PE & CI Juvenile: 0.53 {−0.55, 1.31}; Subadult: −0.88 {−3.15, 0.59}). This was also not the case for adults for IgM or IgG (PE & CI Adult IgG 0.01 {−0.14, 0.16}, Adult IgM 0.04 {−0.31, 0.37}).

We hypothesized the temporal pattern of qRT‐PCR detections events should also show intercohort variation. However, we did not observe clear temporal patterns in infection (Figure 2a), probably because Hendra virus RNA detection was rare with only 4.3% of adults (Bayesian PE, Credible Interval, N: 0.043 {0.033–0.057}, 5/115), 3.4% of juveniles (0.038 {0.018–0.066}, 8/235) and 2.6% of subadults (0.032 {0.011–0.066}, 4/152) testing positive. When we assessed RNA detection using a logistic regression and predicted developmental age (days), we identified no association (PE & CI 0.000 {−0.006, 0.005}; Figure 2b). Furthermore, no cohort had higher odds of being positive for Hendra virus RNA (PE & CI 2016–2.81 {−6.18, 0.187}; 2017–3.59 {−5.18, −2.08}; 2018–4.22{−6.11, −2.61}; 2019–3.78 {−6.27, −1.42}). Furthermore, we found no statistical support for including cohort in our model of qRT‐PCR detection (ELPD‐LOO‐CV estimate ± SE with cohort: −62.5 ± 12.0, without cohort, −61.0 ± 12.5, model difference −1.5 ± 17.5).

### 3.7 Limited variation in adult female anti‐Hendra virus seroprevalence

We restricted our dataset to adult bats and used our Gaussian smoothing process to assess seroprevalence over the longitudinal study. We found limited variation in anti‐Hendra virus IgG and IgM seroprevalence among females (Table 1: a posteriori hypothesis 4 and Figure S12). Furthermore, their seroprevalence levels were comparable to those of males in our dataset.

### 3.8 Temporal patterns of anti‐Hendra virus IgM seroprevalence do not align with IgG, but high cross‐reactivity limits inferences

After establishing that the CM7 mAb could bind *Pteropus* IgM (Supporting Information: Results Section 3), we used these antibodies in our Luminex platform. We categorized the IgM MFI values into ‘IgM seropositive’ and ‘IgM seronegative’ using a gamma mixture model. While a one‐part gamma model fit best, a two‐part gamma mixture model was the next best model (Figure S7D). This provided a seropositivity cutoff value of 1.13 (Figure S7A). Model diagnostics indicated satisfactory convergence, with MCMC trace plots (Figure S7C) and joint posterior distributions supporting model fit (Figure S7E).

When we assessed the temporal dynamics of anti‐Hendra virus IgM seroprevalence in sexually immature birth cohorts we observed no intercohort temporal variation in anti‐Hendra virus IgM seroprevalence (Figure 2a), and including cohort in our model of IgM detection did not improve the model fit (ELPD‐LOO‐CV estimate ± SE cohort model: −247.9 ± 13.9; no cohort model −247.2 ± 14.0; difference − 0.7 ± 19.9). Furthermore, we found no differences by birth cohort in the odds of detecting anti‐Hendra virus IgM antibodies (PE & CI 2016–4.99 {−6.64, −3.51}; 2017–3.46 {−4.12, −2.82}; 2018–3.51 {−4.2, −2.84}; 2019–4.34 {−5.48, −3.28}) (Figure 2b). Like *Bartonella* detection, the odds of anti‐Hendra virus IgM antibodies increased by 0.008 per predicted developmental day (PE and 90% CI: IgM 0.008 {0.006, 0.01}; Figure 2b).

However, our anti‐IgM mAb shows high cross‐reactivity with other antigens on our multiplexed platform (antigens listed in Figure S5E,F). In our PCA analysis, anti‐Hendra virus IgM antibodies did not project with anti‐Nipah virus IgM antibodies on PC2 (10% of variance, PC1: 48.9% of variance) (Figure S5A,F). This indicates that bats possessing anti‐Hendra virus IgM antibodies were not more likely to have IgM antibodies against Nipah virus. This was surprising, as the Nipah virus and Hendra virus G glycoproteins share 83.3% amino acid sequence homology (Harcourt et al., 2000). This PCA result suggests that anti‐Hendra virus IgM levels were not specific to Hendra virus, were likely highly cross‐reactive, and could not provide us with reliable inferences about Hendra virus transmission events. This contrasted with IgG, where anti‐Hendra virus IgG antibodies had a similar projection with anti‐Nipah virus IgG antibodies for both PC1 (31.8% of variance) and PC2 (9.5% of variance; Figure S5C).

## 4 DISCUSSION

Seasonal patterns in pathogen transmission are common in wildlife systems, yet the mechanisms generating these patterns remain difficult to resolve. We evaluated the hypothesis that juveniles drive pulses of Hendra virus transmission when they enter the susceptible pool after losing maternal immunity. We used a cohort‐based, multi‐pathogen design to compare Hendra virus dynamics in juveniles to the dynamics of other pathogens, including *Bartonella* with its known transmission route through close contact. We found no evidence that juvenile cohorts drive winter Hendra virus transmission. The presence of maternal antibodies to Hendra virus varied between cohorts and we did not observe synchronized seroconversion events in juveniles. However, *Bartonella* infections remained consistent across cohorts, with nearly all juveniles acquiring infection within their first year. These findings suggest that, despite synchronized births in *Pteropus* bats and consistent opportunities for transmission, juveniles do not drive shedding pulses of Hendra virus. Other factors, such as environmental stressors, require further exploration as potential drivers of seasonal shedding pulses of Hendra virus. More broadly, this comparative, multi‐pathogen framework offers a generalizable approach for wildlife disease systems in which environmental conditions, host demography and immunity shift concurrently, creating overlapping seasonal processes that otherwise remain difficult to disentangle.

Testing the juvenile‐driver hypothesis required first understanding how maternally derived antibodies varied among birth cohorts. Although maternal antibody transfer is often assumed to be consistent (Garnier et al., 2012; Peel et al., 2014), it can be influenced by environmental and genetic factors that affect placental and mammary transfer (Boulinier & Staszewski, 2008; Grindstaff et al., 2003). Our 4‐year study revealed interannual variation in maternal anti‐Hendra virus IgG. The 2019 cohort, sampled following a documented food shortage, showed particularly reduced early‐life antibody levels, suggesting that energy limitation may have constrained antibody transfer. While we could not directly measure dam–pup correlations because dependent pups were not sampled, strong correlations have been documented previously (Baker et al., 2014; Brook et al., 2019; Peel et al., 2018). It remains unclear if the absence of high titers in young juveniles from certain cohorts reflects reduced maternal transfer or unusually rapid waning. Additional cohorts and more frequent sampling of early‐age juveniles will be needed to resolve the mechanisms behind this variation.

Juvenile bats with higher anti‐Hendra virus IgG were more likely to test positive for viral RNA, indicating that elevated IgG in this age class reflects recent or ongoing infection rather than past exposure. The link between Hendra virus RNA detection and IgG levels in juveniles, despite the low overall prevalence of viral RNA detection (2.6%–4.3%), consistent with other Hendra virus studies (Edson et al., 2019; Field et al., 2011), provides empirical support for using IgG dynamics to infer juvenile transmission events. The absence of this relationship in adults likely reflects their more complex exposure histories, repeated boosting and potentially antibody‐mediated viral suppression rather than clearance, as observed for other bat viruses (Schuh et al., 2017).

Although we validated an anti‐feline IgG monoclonal antibody for Pteropus IgM, the IgM measurements lacked pathogen specificity. High cross‐reactivity of IgM across viral antigens, consistent with challenges described for other wildlife immunological assays (Crowley et al., 2024; Flies, 2020; Flies et al., 2012), prevented IgM from serving as an indicator of recent Hendra virus infection. Bat IgM measurements may still be valuable for assessing general immune investment, but pathogen‐specific inferences will require more reagent development and optimization.

*Bartonella* infections increased predictably across all juvenile cohorts, consistent with transmission via wingless nycteribiid bat flies and with previous findings that pups are born uninfected and acquire *Bartonella* during their first year of life (Fagre et al., 2023; McKee et al., 2021). Because *Bartonella* transmission requires close contact, stable *Bartonella* dynamics across years suggest that basic opportunities for horizontal transmission were consistent across cohorts. This consistency contrasts with the highly variable Hendra virus IgG patterns, implying that shifts in maternal immunity or other environmental factors—not changes in contact rates or cohort demography—drive variation in Hendra virus serological dynamics. While the two pathogens differ in chronicity (*Bartonella* can persist, whereas Hendra virus infections may be short or intermittently shedding; Plowright et al., 2016), our focus on naïve juveniles minimizes differences in interpretability during the first year of life.

Several limitations should be acknowledged when interpreting our findings. Sampling every 2–3 months likely missed narrow windows of maternal antibody waning and initial seroconversion, and our inability to sample dependent pups constrained our ability to confirm dam–pup transfer directly. Species‐specific differences between *P. alecto* and *P. poliocephalus* appeared minimal, though subtle variation cannot be excluded. Future work should incorporate more frequent sampling of early‐age juveniles, direct sampling of mother–pup pairs and improved immunological tools designed for *Pteropus* species. Long‐term mark–recapture could clarify reinfection and antibody‐boosting dynamics, and coupling serological data with quantitative environmental metrics would help identify drivers of maternal antibody variation.

## 5 CONCLUSIONS

Through systematic hypothesis testing and a multi‐pathogen approach, we demonstrate that seasonal birth pulses alone cannot explain Hendra virus transmission patterns in Australian flying foxes. The combination of interannual variation in maternal antibody transfer, lack of synchronized seroconversion and consistent bacterial transmission across cohorts indicates that demographic forcing is not the dominant driver of Hendra virus winter pulses. These findings have implications for disease prediction and management—models based solely on host demography may fail to capture transmission dynamics in systems where maternal immunity varies and environmental factors modulate transmission probability. More broadly, our work demonstrates the value of comparative approaches that use multiple pathogens to disentangle the complex drivers of seasonal disease dynamics. As we face increasing challenges from emerging zoonoses, understanding this complexity becomes essential for developing prediction and intervention strategies.

## AUTHOR CONTRIBUTIONS

*Conceptualization*: Daniel E. Crowley, Caylee A. Falvo, Chris K. Grant, Tamika J. Lunn, Devin N. Jones, Benny Borremans, Daniel J. Becker, Manuel Ruiz‐Aravena, Vincent J. Munster, Agnieszka Rynda‐Apple, Alison J. Peel and Raina Plowright. *Methodology*: Daniel E. Crowley, Caylee A. Falvo, Chris K. Grant, Trenton Bushmaker, Evelyn Benson, Benny Borremans, Daniel J. Becker, Clifton D. McKee, Eric D. Laing, Laura B. Goodman, Vincent J. Munster, Agnieszka Rynda‐Apple and Alison J. Peel. *Software*: Daniel E. Crowley and Clifton D. McKee. *Validation*: Daniel E. Crowley, Caylee A. Falvo, Chris K. Grant, Trenton Bushmaker, Evelyn Benson, Clifton D. McKee, Vincent J. Munster and Alison J. Peel. *Formal analysis*: Daniel E. Crowley, Chris K. Grant, Benny Borremans, Clifton D. McKee, Agnieszka Rynda‐Apple and Alison J. Peel. *Investigation*: Daniel E. Crowley, Caylee A. Falvo, Chris K. Grant, Tamika J. Lunn, Devin N. Jones, Trenton Bushmaker, Adrienne S. Dale, Evelyn Benson, Benny Borremans, Daniel J. Becker, Y. Tina Yu, Manuel Ruiz‐Aravena, Michelle Michie, Ina L. Smith, Laura B. Goodman, Vincent J. Munster, Agnieszka Rynda‐Apple, Alison J. Peel and Raina Plowright. *Resources*: Daniel E. Crowley, Chris K. Grant, Daniel J. Becker, Eric D. Laing, Christopher C. Broder, Lianying Yan, Spencer L. Sterling, Ina L. Smith, Laura B. Goodman, Vincent J. Munster, Agnieszka Rynda‐Apple, Alison J. Peel, Raina Plowright and Rasa Petraityte‐Burneikiene. *Data curation*: Daniel E. Crowley, Caylee A. Falvo, Tamika J. Lunn, Devin N. Jones, Manuel Ruiz‐Aravena and Alison J. Peel. *Writing—original draft*: Daniel E. Crowley, Caylee A. Falvo, Manuel Ruiz‐Aravena, Agnieszka Rynda‐Apple, Alison J. Peel and Raina Plowright. *Writing—review and editing*: Daniel E. Crowley, Caylee A. Falvo, Chris K. Grant, Tamika J. Lunn, Devin N. Jones, Trenton Bushmaker, Evelyn Benson, Adrienne S. Dale, Benny Borremans, Daniel J. Becker, Y. Tina Yu, Manuel Ruiz‐Aravena, Eric D. Laing, Christopher C. Broder, Spencer L. Sterling, Michelle Michie, Ina L. Smith, Laura B. Goodman, Vincent J. Munster, Agnieszka Rynda‐Apple, Alison J. Peel and Raina Plowright. *Visualization*: Daniel E. Crowley, Caylee A. Falvo, Chris K. Grant, Clifton D. McKee, Ina L. Smith, Vincent J. Munster and Raina Plowright. *Supervision*: Manuel Ruiz‐Aravena, Ina L. Smith, Laura B. Goodman, Vincent J. Munster, Agnieszka Rynda‐Apple, Alison J. Peel and Raina Plowright. *Project administration*: Daniel E. Crowley, Chris K. Grant, Tamika J. Lunn, Devin N. Jones, Trenton Bushmaker, Adrienne S. Dale, Daniel J. Becker, Manuel Ruiz‐Aravena, Ina L. Smith, Laura B. Goodman, Vincent J. Munster, Agnieszka Rynda‐Apple, Alison J. Peel and Raina Plowright. *Funding acquisition*: Chris K. Grant, Daniel J. Becker, Laura B. Goodman, Vincent J. Munster, Agnieszka Rynda‐Apple, Alison J. Peel and Raina Plowright.

## FUNDING INFORMATION

The project was supported by the National Science Foundation (DEB1716698, EF2133763), and the DARPA PREEMPT programme Cooperative Agreement # D18AC00031. The content of the information does not necessarily reflect the position or the policy of the U.S. government, and no official endorsement should be inferred. A.J.P. was supported by an ARC DECRA fellowship (DE190100710) and a Queensland Government Accelerate Postdoctoral Research Fellowship. *Bartonella* analyses were supported by a grant from the Royal Society for Tropical Medicine and Hygiene to D.J.B.

## CONFLICT OF INTEREST STATEMENT

The authors declare no conflict of interest.

## Supporting information


**Figure S1.** A flow diagram of the sample sizes in each analysis and figure. The restriction criteria (blue) for the analyses impacted the total sample size (purple) for each plot and analysis.
**Figure S2.** Bat morphological measurements and Bayesian mixture model analysis across birth cohorts.
**Figure S3.** Comparison of Bayesian mixture model performance between all bat species versus single‐species analysis.
**Figure S4.** Validation of Anti‐IgM Antibody (A) Pteropus serum was size fractionated into 44 collection tubes using an S‐300 column.
**Figure S5.** Principal components analysis (PCA) results comparing IgM and IgG antibodies.
**Figure S6.** IgG Diagnostics: (A) A histogram of anti‐Hendra virus IgG lnMFI. Overlaid are the posterior distributions from the Bayesian gamma mixture model indicating seronegative and seropositive distributions. The orange line is 2 SD above the mean of the seronegative distribution, and the yellow line is 3 SD above the mean of the seronegative distribution. (B). A plot of anti‐Hendra virus IgG lnMFI and the probability of being classified as seropositive by the mixture distribution. The red dashed line is the 50% cutoff for the two distributions, the orange line is 2 SD above the mean of the seronegative distribution, and the yellow line is 3 SD above the mean of the seronegative distribution (C) The MCMC trace plots from the mixture distribution model. (D) Loo statistics comparing the gamma mixture model to three competitor models (E) The histogram and joint posteriors from the model. Theta is the parameter for the probability of belonging to the seropositive or seronegative group. Alpha and beta parameters describe the two gamma distributions.
**Figure S7.** IgM Model Diagnostics: (A) A histogram of lnMFI of IgM antibodies binding the Hendra virus glycoprotein. Overlaid are the posterior distributions from the Bayesian gamma mixture model indicating seronegative and seropositive distribution. The red dashed line is the 50% cutoff for the two distributions, the orange line is 2 SD above the mean of the seronegative distribution, and the yellow line is 3 SD above the mean of the seronegative distribution (B) A plot of anti‐Hendra virus IgM lnMFI and the probability of being classified as seropositive by the mixture distribution. The red dashed line is the 50% cutoff for the two distributions, the orange line is 2 SD above the mean of the seronegative distribution, and the yellow line is 3 SD above the mean of the seronegative distribution (C) The MCMC trace plots from the mixture distribution model. (D) Loo statistics comparing the gamma mixture model to three competitor models (E) The histogram and joint posteriors from the model. Theta is the parameter for the probability of belonging to the seropositive or seronegative group. Alpha and beta parameters describe the two gamma distributions.
**Figure S8.** Details on seroprevalence and prevalence dynamics models. (A) Basic model structure used to estimate prevalence dynamics in relation to developmental age since initial cohort sampling. (B) Cohort specific posterior estimates for a, rho, and alpha. Red line indicates 0. Black dashed line indicates posterior mean.
**Figure S9.** Model output for serological and pathogen detection patterns, including juvenile bat cohort prevalence dynamics of (A) anti‐Hendra virus IgG, (B), anti‐Hendra virus IgM, (C) Hendra virus RNA, (D) Bartonella spp. and (E) anti‐Menangle virus‐N IgG.
**Figure S10.** Phylogenetic relationships between Bartonella gltA sequences.
**Figure S11.** Menangle Virus Antigen IgG Diagnostics: (A, B) A histogram of anti‐Menangle virus N (A) & HN (B) IgG antibodies natural log MFI. Overlaid are the posterior distributions from the Bayesian gamma mixture model (A) and normal mixture model (B) indicating seronegative and seropositive distributions. The orange line is 2 SD above the mean of the seronegative distribution, and the yellow line is 3 SD above the mean of the seronegative distribution. (C, D) Model comparison diagnostics for the mixture distributions of MenV‐N (C) and MenV‐HN (D). The best fitting model for the anti‐N protein (C) was a two‐part gamma mixture distribution. The best fitting model for the anti‐HN protein (D) was a one‐part gamma mixture distribution.
**Figure S12.** Adult Pteropus bat anti‐Hendra virus serological dynamics.
**Figure S13.** Seroprevalence and PCR prevalence dynamics from the Gaussian smoothing model. (A) Analysis restricted to bats with >95% posterior probability of cohort assignment. (B) Analysis including all bats from panel A, but without their cohort stratification. (C) Model comparison (ELPD LOO) contrasting cohort‐stratified and cohort‐naive models. In the figure, IgG and IgM seroprevalence refers to HeV seroprevalence. Shaded regions in panels A and B represent 95% credible intervals.
**Figure S14.** Seroprevalence patterns when dataset is restricted to Redcliffe and Toowoomba, the two sites that were continuously sampled throughout the longitudinal study.
**Figure S15.** (A) IgG and IgM dynamics with increasingly stringent integer value cutoffs for lnMFI cutoff to establish seropositivity (1 to 4).

**Table S1.** Prevalence of *Bartonella* in bat blood spot FTA cards across demographic variables. Continuously sampled sites included Redcliffe and Toowoomba (both sampled in 2018, 2019 and 2020). Nomadic sites included Gympie (2019), Hervey Bay (2018 and 2020), Maclean (2018) and Mount Ommaney (2019).

## Data Availability

Data available from Zenodo https://doi.org/10.5281/zenodo.15982445 (Crowley et al., 2025). *Bartonella* gltA sequences have been deposited in GenBank (accession numbers PX739495–PX739549).
