# Supplementary material for: Cohorts of immature Pteropus bats show interannual variation in Hendra virus serology
Source: J Anim Ecol. 2026 Feb 1;95(3):521–37. doi: 10.1111/1365-2656.70213 (PMC12957713; doi:10.1111/1365-2656.70213)
Supplement: Supplementary file 1 — Figure S1. A flow diagram of the sample sizes in each analysis and figure. The restriction criteria (blue) for the analyses impacted the total sample size (purple) for each plot and analysis. Figure S2. Bat morphological measurements and Bayesian mixture model analysis across birth cohorts. Figure S3. Comparison of Bayesian mixture model performance between all bat species versus single‐species analysis. Figure S4. Validation of Anti‐IgM Antibody (A) Pteropus serum was size fractionated into 44 collection tubes using an S‐300 column. Figure S5. Principal components analysis (PCA) results comparing IgM and IgG antibodies. Figure S6. IgG Diagnostics: (A) A histogram of anti‐Hendra virus IgG lnMFI. Overlaid are the posterior distributions from the Bayesian gamma mixture model indicating seronegative and seropositive distributions. The orange line is 2 SD above the mean of the seronegative distribution, and the yellow line is 3 SD above the mean of the seronegative distribution. (B). A plot of anti‐Hendra virus IgG lnMFI and the probability of being classified as seropositive by the mixture distribution. The red dashed line is the 50% cutoff for the two distributions, the orange line is 2 SD above the mean of the seronegative distribution, and the yellow line is 3 SD above the mean of the seronegative distribution (C) The MCMC trace plots from the mixture distribution model. (D) Loo statistics comparing the gamma mixture model to three competitor models (E) The histogram and joint posteriors from the model. Theta is the parameter for the probability of belonging to the seropositive or seronegative group. Alpha and beta parameters describe the two gamma distributions. Figure S7. IgM Model Diagnostics: (A) A histogram of lnMFI of IgM antibodies binding the Hendra virus glycoprotein. Overlaid are the posterior distributions from the Bayesian gamma mixture model indicating seronegative and seropositive distribution. The red dashed line is the 50% cutoff for the two distribu [file JANE-95-521-s001.docx]

|  |
| --- |
| Supp. Fig. 1: A flow diagram of the sample sizes in each analysis and figure. The restriction criteria (blue) for the analyses impacted the total sample size (purple) for each plot and analysis. |

| \|  \| \| --- \| \| **Supp. Fig. 2 Bat morphological measurements and Bayesian mixture model analysis across birth cohorts.**  **(A)** Bat weight measurements over time (2017-2021) showing observed data points (orange), simulated data (green), and adult filtered outliers (green). Gray shaded regions indicate a food shortage event. Black gaussian distributions at bottom represent the assumed birth pulse event for *BFF* bats. Asterisk indicates distance between assumed birth pulse and earliest catching session where dependent pups were caught and measured. **(B)** Pairwise relationships and distributions of morphological measurements. Scatter plots show correlations between weight and forearm length (left panels), with marginal histograms displaying individual measurement distributions. Also shown are the transformations of weight and forearm that were used in the cohort model. **(C)** Prior probability distributions for Dirichlet mixture model coefficients for birth cohort assignments (cohorts 1-5, indicated by color). Upper panel shows overall cohort probability densities, lower panels show coefficient-specific prior distributions for FA std:sex F, FA std:sex M, WT std:sex F, and WT std:sex M parameters. (**D)** Full Cohort Model **(E)** Posterior distributions from Bayesian analysis. Upper panel displays posterior birth cohort probabilities (i.e. probability a given bat belonged to a specific cohort). Lower panels show posterior coefficient distributions for weight (WT) and forearm (FA) by sex, with μ values indicated by vertical dashed lines. \|  \|  \|  \| \| --- \| --- \| \| Supp. Fig. 3: **Comparison of Bayesian mixture model performance between bat species.** In the figure we show posterior density distributions for model parameters comparing analyses using black flying fox (BFF) only data and grey headed flying fox (GHFF) only data.. **Top panel:** Posterior distributions of growth model coefficients for weight and forearm measurements by sex, comparing the model (BFF shown in red/orange and GHFF shown in teal). The overlapping distributions indicate that including multiple species does not substantially impact parameter estimates for weight and forearm measurements across male and female bats. **Bottom panel:** Hendra virus (HeV) IgG mixture model density plots showing the fitted gamma mixture distributions for seronegative (blue dashed line) and seropositive (red dashed line) components, with the total mixture distribution (black solid line) overlaid on histograms of observed log-transformed IgG MFI values. Vertical red dashed lines indicate optimal diagnostic cutoff values calculated at 95% specificity (GHFF Only: 2.55; BFF Only: 2.34). The similar distribution shapes and cutoff values between the two analyses demonstrate that restricting the analysis to a single bat species yields comparable results to the pooled-species approach, validating the mixture modeling framework for serological data analysis. \|  \|  \| 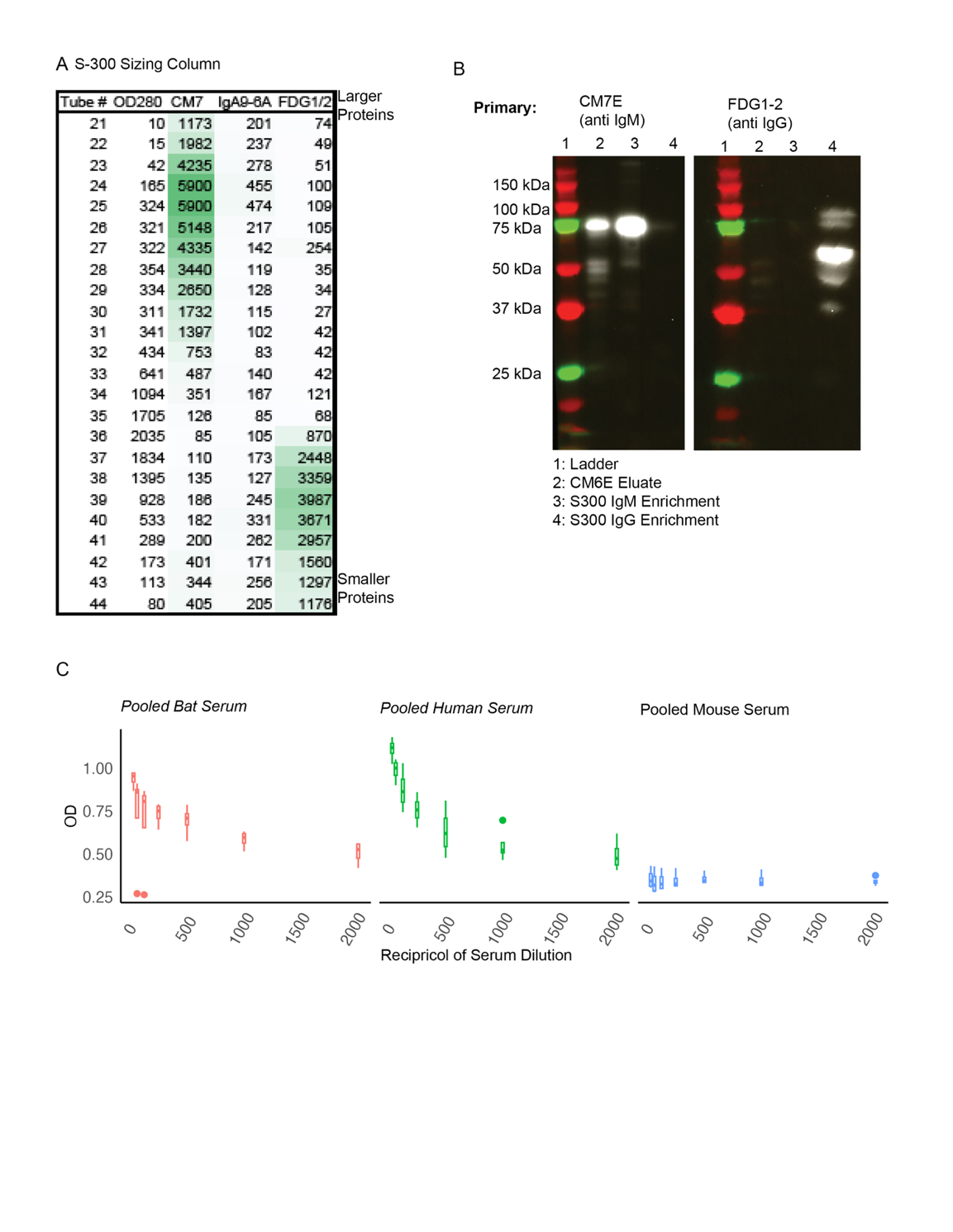 \| \| --- \| \| Supp. Fig. 4: Validation of Anti-IgM Antibody (A) *Pteropus* serum was size fractionated into 44 collection tubes using an S-300 column. Each fraction was continuously monitored and an OD280 reading was taken. Each collection tube was then tested by direct ELISA using three mAbs: CM7, IgA9-6A, and FDG1-2. The OD450 of these ELISA reactions are listed in the table. Darker green shading in a cell indicates stronger binding. CM7 bound larger protein eluates (tubes 23-31), while FDG1-2 is bound to smaller protein eluates (tubes 37-42). IgA9-6A did not clearly recognize any fraction. (B) Western blot using antibodies CM7 and FDG1-2 to probe fractionated *Pteropus* serum. Antibodies were probed against three unique protein preparations. Lane 1 is the serum affinity purified using a CM6E (another feline anti-IgM antibody) antibody column. Lane 2 is the serum enriched for IgM sized proteins using an S-300 column. Lane 3 is the serum enriched for IgG sized proteins using an S-300 column. CM7 binds a 75 kDa protein in the CM6E affinity column eluate and the S-300 IgM size-fractionated proteins. \|  \| 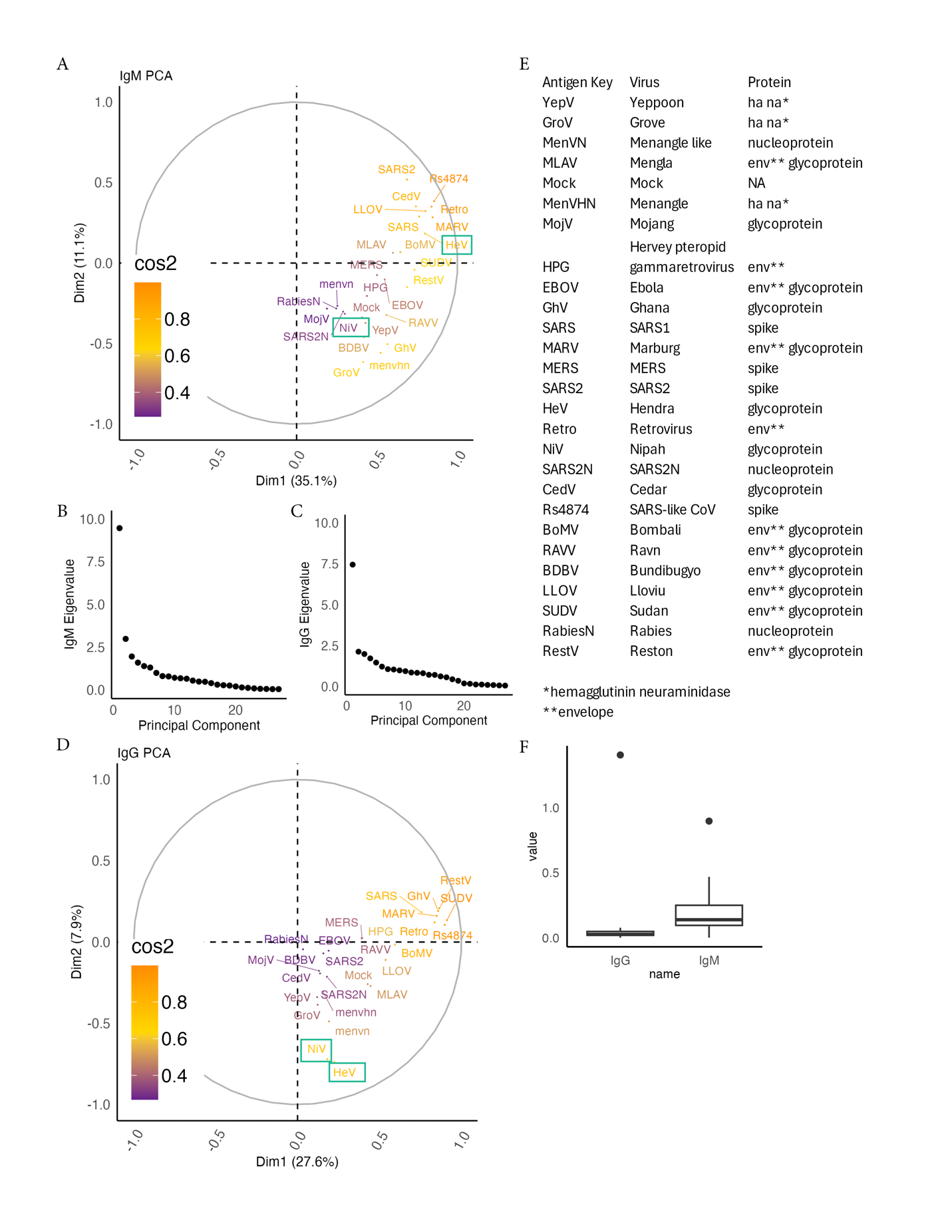 \|  \| \| --- \| --- \| \| Supp. Fig. 5: Principal components analysis (PCA) results comparing IgM and IgG antibodies. The Luminex platform contained over 20 antigens. The PCA assesses correlations of all antigens in full dataset (A,D). Variables are colored by squared cosine distance (cos2) value. Larger cos2 values indicate the antigen is better explained by these two components (Dim2 and Dim1) compared to an antigen with a low cos2 value. (B,C) Scree plots of eigenvalues from the principal component’s decomposition. E. A list of Luminex antigens tested in the PCA. For Hendra, Nipah, Cedar, Ghana and Mòjiāng virus, glycoprotein refers to the envelope attachment glycoprotein. For Zaire ebolavirus, Bundibugyo ebolavirus, Bombali ebolavirus, Marburg marburgvirus, and Ravn virus envelope glycoprotein refers to the soluble trimeric fusion glycoprotein (GP) ectodomains. For severe acute respiratory syndrome 1 & 2, Middle East respiratory syndrome coronaviruses, spike refers to the spike glycoprotein trimer. For Menangle virus and Rabies, nucleoprotein refers to the structural nucleoprotein. (F) The box plot compares the distribution of absolute differences in principal component loadings between Hendra virus and Nipah antigens for IgG and IgM responses. Smaller differences indicate less cross-reactivity (more similar response patterns in PCA space). The analysis demonstrates that IgM responses show significantly greater cross-reactivity between these paramyxoviruses compared to IgG responses. \|  \| |
| --- | --- | --- | --- | --- | --- | --- | --- | --- | --- | --- | --- | --- |
|  |
|  |

| 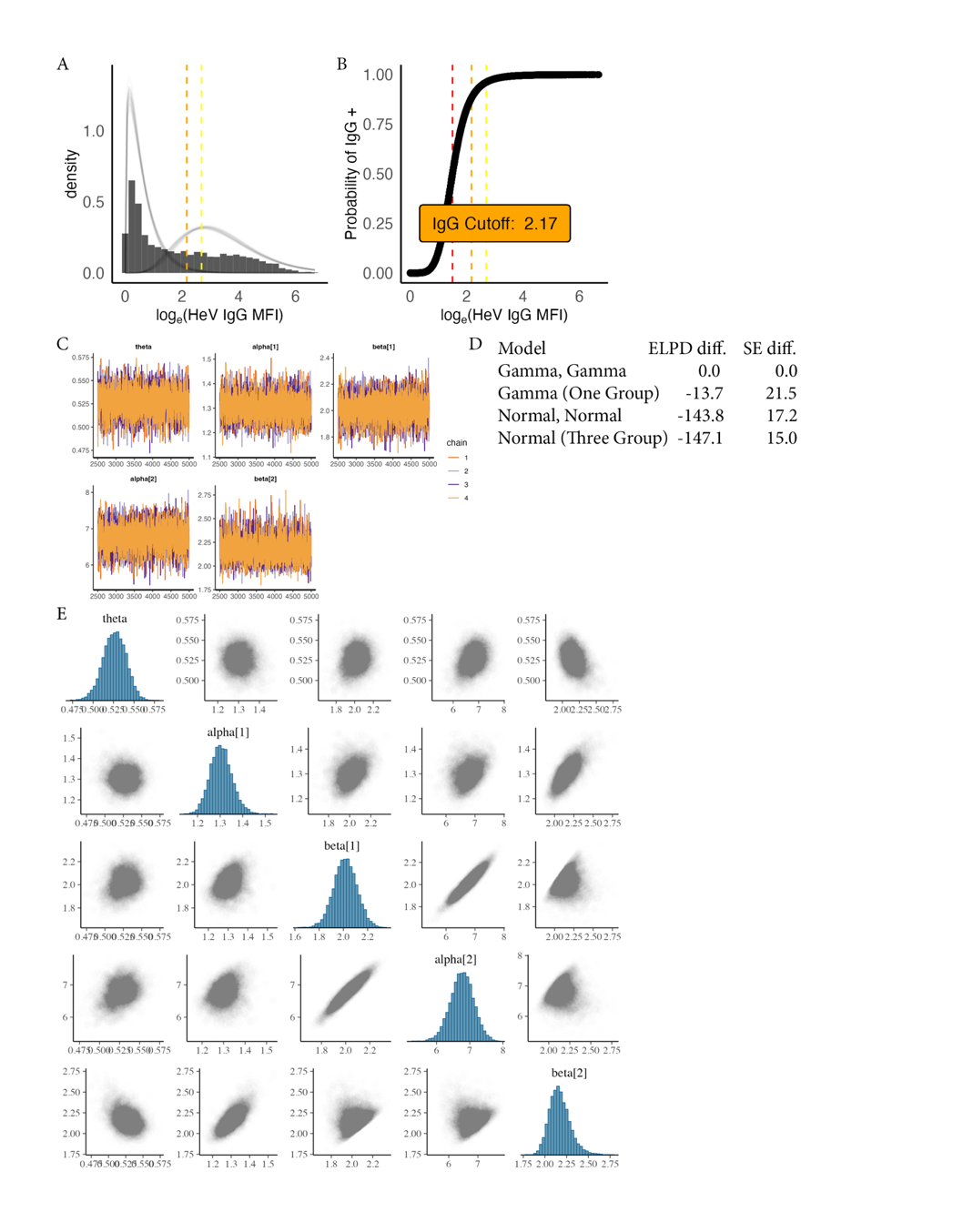 |
| --- |
| Supp. Fig. 6: IgG Diagnostics: (A) A histogram of anti-Hendra virus IgG lnMFI. Overlaid are the posterior distributions from the Bayesian gamma mixture model indicating seronegative and seropositive distributions. The orange line is 2 SD above the mean of the seronegative distribution, and the yellow line is 3 SD above the mean of the seronegative distribution. (B). A plot of anti-Hendra virus IgG lnMFI and the probability of being classified as seropositive by the mixture distribution. The red dashed line is the 50% cutoff for the two distributions, the orange line is 2 SD above the mean of the seronegative distribution, and the yellow line is 3 SD above the mean of the seronegative distribution (C) The MCMC trace plots from the mixture distribution model. (D) Loo statistics comparing the gamma mixture model to three competitor models (E) The histogram and joint posteriors from the model. Theta is the parameter for the probability of belonging to the seropositive or seronegative group. Alpha and beta parameters describe the two gamma distributions. |

| 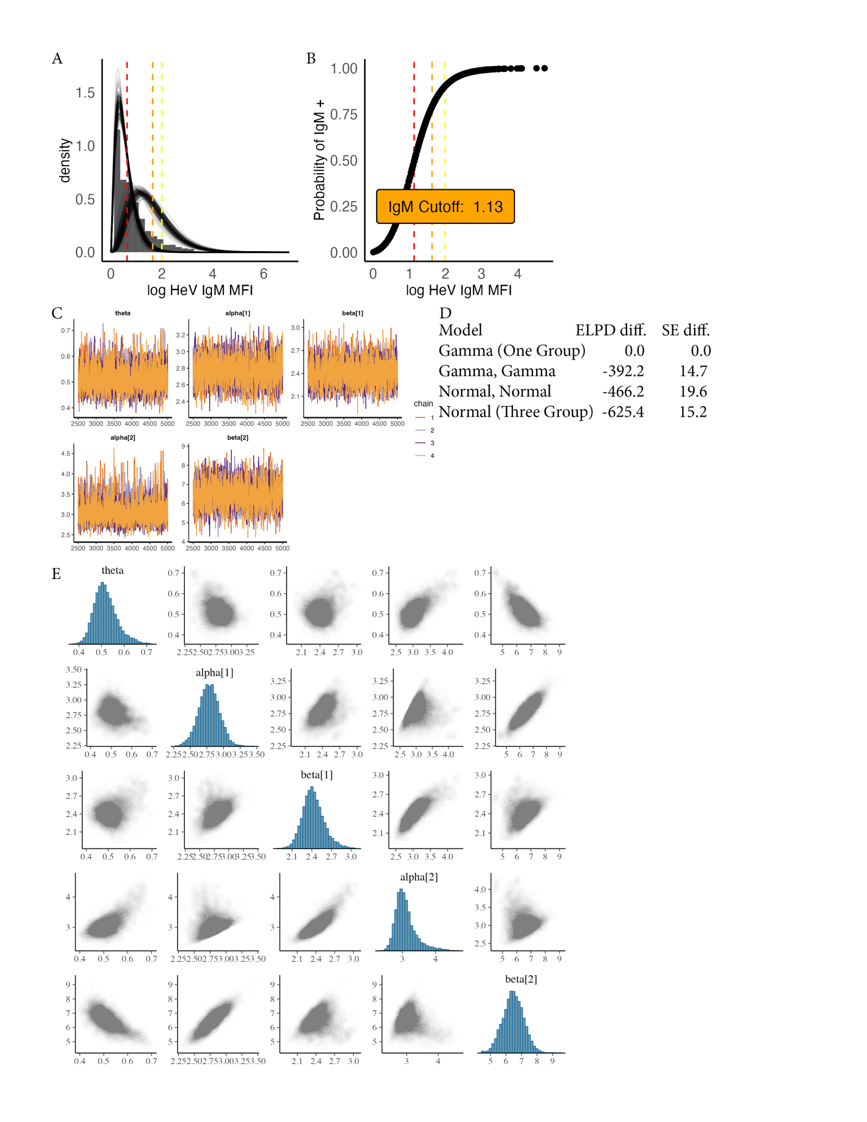 |
| --- |
| Supp. Fig. 7: IgM Model Diagnostics: (A) A histogram of lnMFI of IgM antibodies binding the Hendra virus glycoprotein. Overlaid are the posterior distributions from the Bayesian gamma mixture model indicating seronegative and seropositive distribution. The red dashed line is the 50% cutoff for the two distributions, the orange line is 2 SD above the mean of the seronegative distribution, and the yellow line is 3 SD above the mean of the seronegative distribution (B) A plot of anti-Hendra virus IgM lnMFI and the probability of being classified as seropositive by the mixture distribution. The red dashed line is the 50% cutoff for the two distributions, the orange line is 2 SD above the mean of the seronegative distribution, and the yellow line is 3 SD above the mean of the seronegative distribution (C) The MCMC trace plots from the mixture distribution model. (D) Loo statistics comparing the gamma mixture model to three competitor models (E) The histogram and joint posteriors from the model. Theta is the parameter for the probability of belonging to the seropositive or seronegative group. Alpha and beta parameters describe the two gamma distributions. |

|  |
| --- |
| Supp. Fig. 8: Details on seroprevalence and prevalence dynamics models. (A) Basic model structure used to estimate prevalence dynamics in relation to developmental age since initial cohort sampling. (B) Cohort specific posterior estimates for a, rho, and alpha. Red line indicates 0. Black dashed line indicates posterior mean. |
| 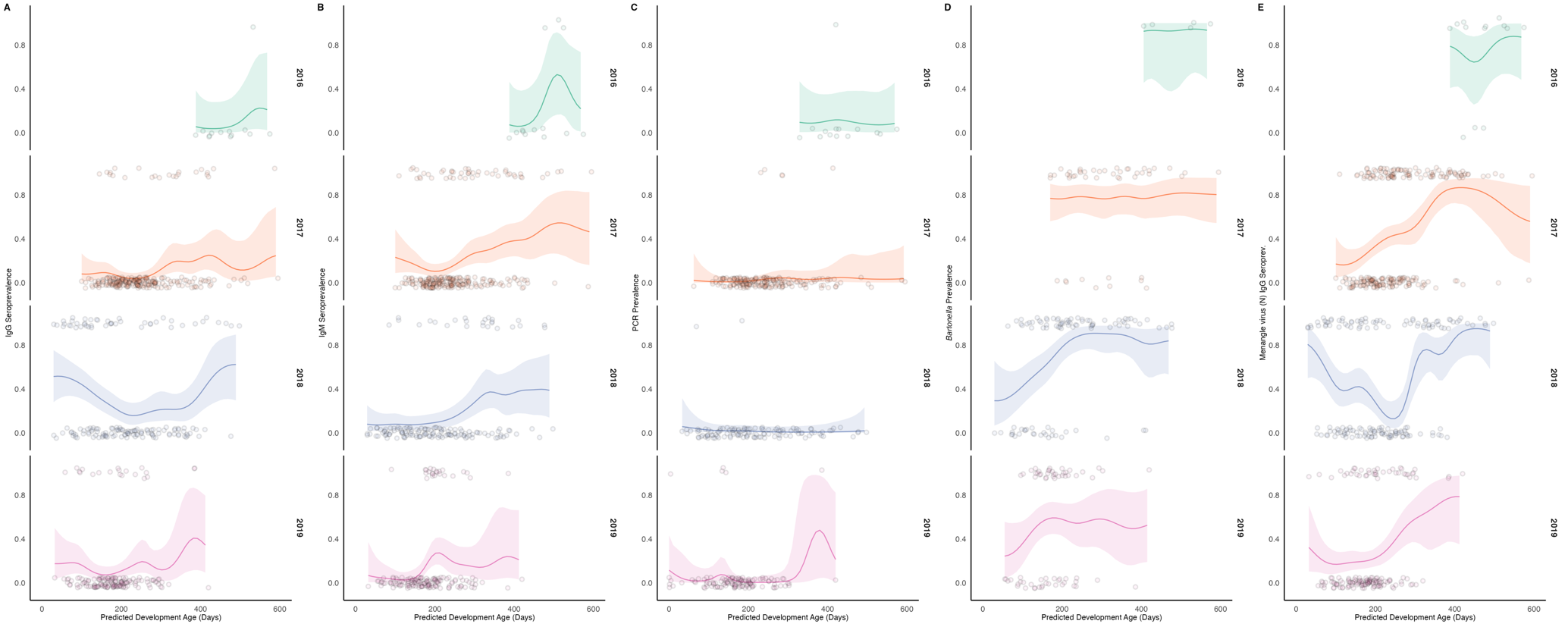 |
| Supp. Fig. 9: Model output for serological and pathogen detection patterns, including juvenile bat cohort prevalence dynamics of (A) anti-Hendra virus IgG, (B), anti-Hendra virus IgM, (C) Hendra virus RNA, (D) *Bartonella* spp. and (E) anti-Menangle virus-N IgG. On the X axis is predicted developmental age , in days, since initial cohort sampling. The estimates are from our Gaussian smoothing model. The shaded region around the prevalence estimate represents the 95% credible interval from the Gaussian smoothing model. Data is faceted by cohort classification labels (i.e. 2016, 2017, 2018, 2019), which are based on the year the bat was predicted to be born. Data points from each cohort are also shown. Points are jittered on the x axis to show overlapping observations. |

| 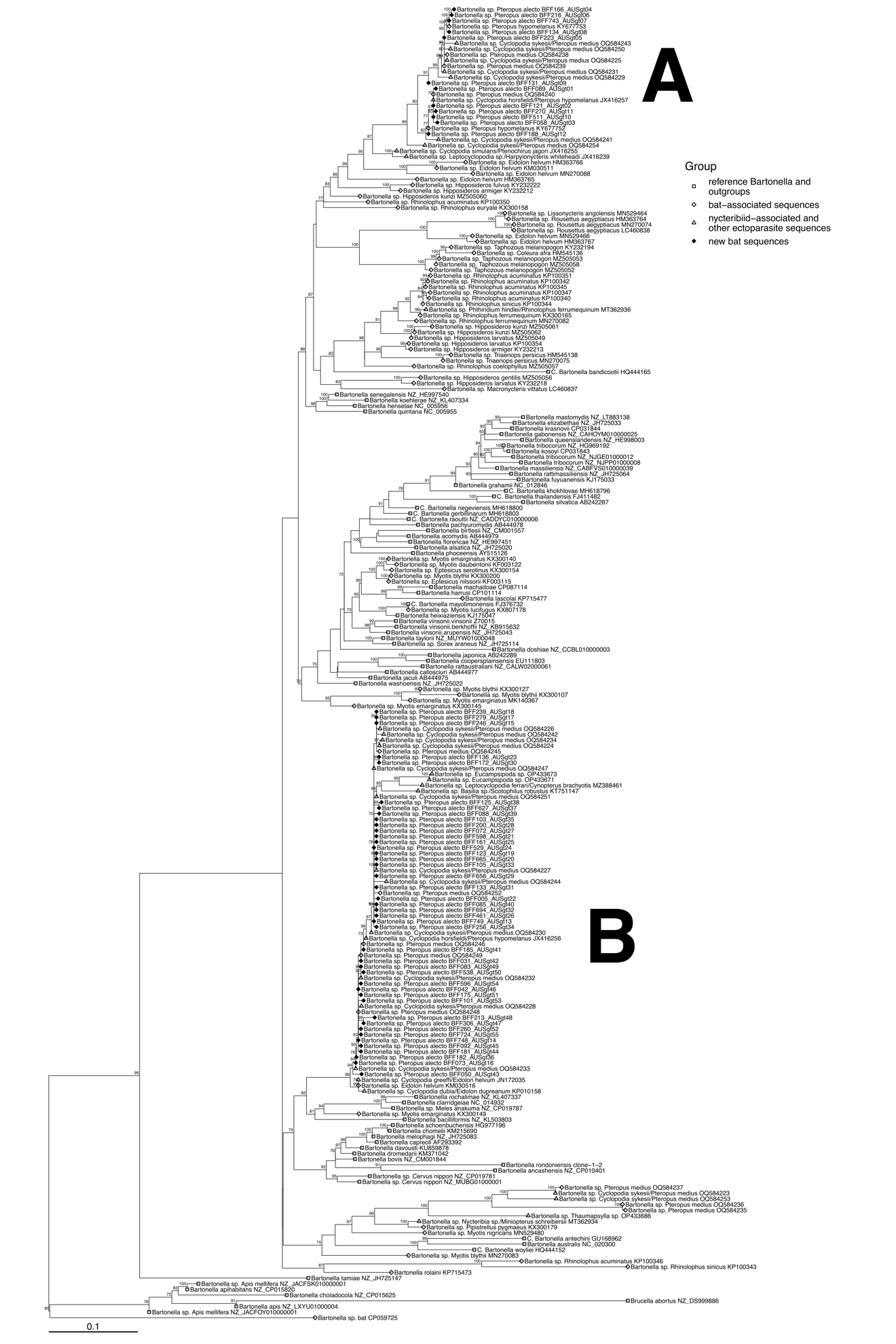 |
| --- |
| Supp. Fig. 10: Phylogenetic relationships between *Bartonella* *gltA* sequences. Separate groups, including new sequences detected in *Pteropus* bats in Australia, are indicated by distinct symbols. The maximum likelihood tree was inferred using a GTR+F+R6 model in IQ-TREE from a 337 bp alignment. Numbers next to nodes indicate the percent bootstrap support after 1000 replicates. Branch lengths are in units of  substitutions per site. |

|  |
| --- |
| Supp. Fig. 11: Menangle Virus Antigen IgG Diagnostics: (A-B) A histogram of anti-Menangle virus N (A) & HN (B) IgG antibodies natural log MFI. Overlaid are the posterior distributions from the Bayesian gamma mixture model (A) and normal mixture model (B) indicating seronegative and seropositive distributions. The orange line is 2 SD above the mean of the seronegative distribution, and the yellow line is 3 SD above the mean of the seronegative distribution. (C-D) Model comparison diagnostics for the mixture distributions of MenV-N (C) and MenV-HN (D). The best fitting model for the anti-N protein (C) was a two-part gamma mixture distribution. The best fitting model for the anti-HN protein (D) was a one-part gamma mixture distribution. To establish a cutoff, the next best fitting model (two-part normal mixture distribution) was chosen. E. Juvenile bat cohort prevalence dynamics of anti-Menangle virus HN IgG. On the X axis is developmental age since initial cohort sampling in each year (December). The estimates are from our Gaussian smoothing model. The shaded region around the prevalence estimate represents the 95% credible interval from the Gaussian smoothing model. Cohort classification (i.e. 2016, 2017, 2018, 2019) is based on the year the bat was born. |
| 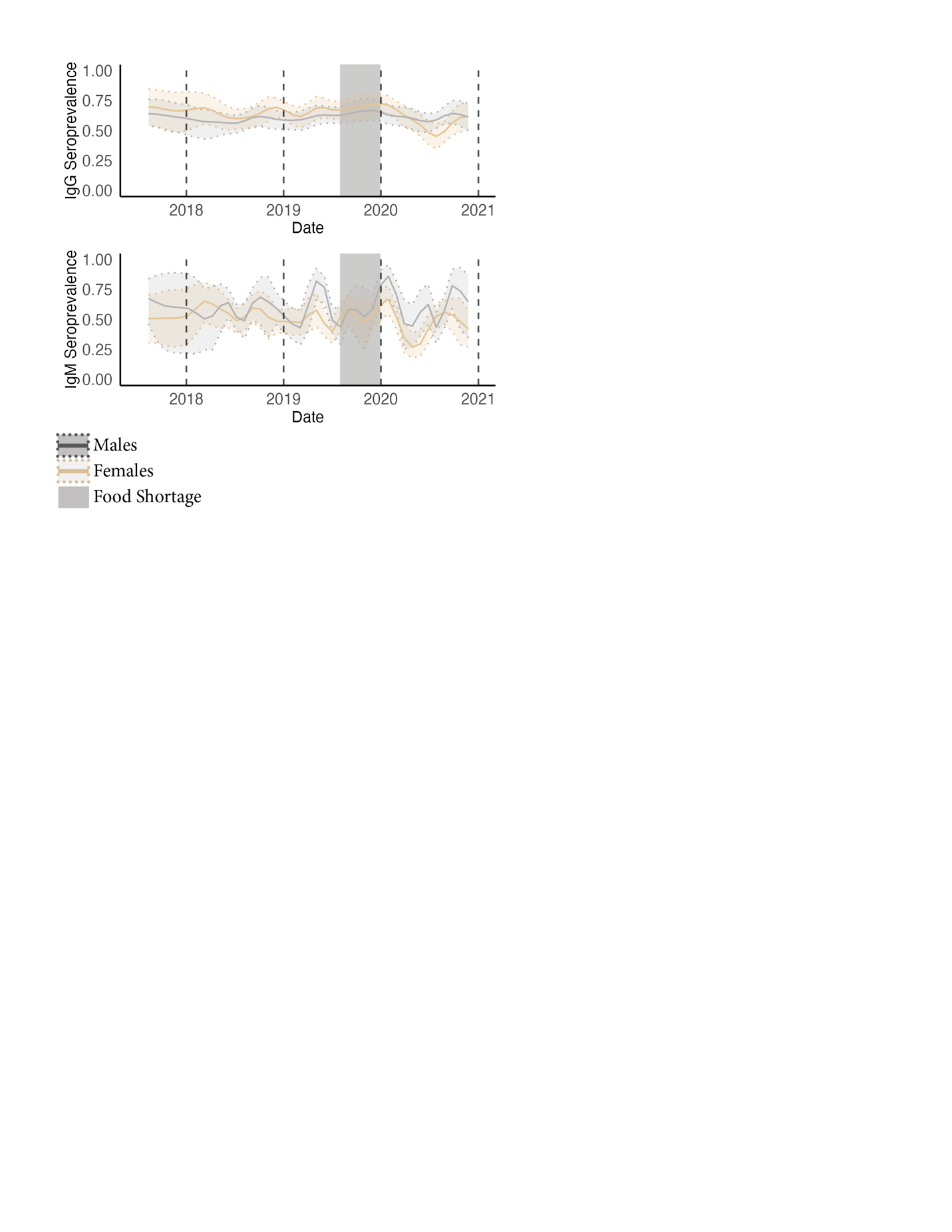 |
| Supp. Fig. 12: Adult *Pteropus* bat anti-Hendra virus serological dynamics. The shaded region around the prevalence estimate represents the 95% credible interval from the Gaussian smoothing model. In light grey is a food shortage event. |

|  |
| --- |
| Supp. Fig. 13: Seroprevalence and PCR prevalence dynamics from the Gaussian smoothing model. (A) Analysis restricted to bats with >95% posterior probability of cohort assignment. (B) Analysis including all bats from panel A, but without their cohort stratification. (C) Model comparison (ELPD LOO) contrasting cohort-stratified and cohort-naive models. In the figure, IgG and IgM seroprevalence refers to HeV seroprevalence. Shaded regions in panels A and B represent 95% credible intervals. |

|  |
| --- |
| Supp. Fig. 14: Seroprevalence patterns when dataset is restricted to Redcliffe and Toowoomba, the two sites that were continuously sampled throughout the longitudinal study. (A) The seroprevalence patterns of anti-Hendra virus IgM & IgG, faceted by the two sampling locations. The estimates are from our Gaussian smoothing model. The shaded region around the prevalence estimate represents the 95% credible interval from the Gaussian smoothing model. (B) Results from a logistic regression predicting the IgG or IgM seroprevalence (dependent binary variables). The independent variables are the predicted birth cohort (2017, 2018, & 2019), the sampling location (Toowoomba or Redcliffe), and the continuous variable developmental age since initial cohort sampling. The plots are the posterior distributions of the odds ratios from this logistic regression. The logistic regression does not include the 2016 cohort due to its low sample size. For the odds ratios, the reference cohort is the 2017 cohort and the reference sampling location is Redcliffe. For both IgM and IgG, the posterior distribution for the odds ratio of Toowoomba contains 0, indicating no differences in IgM and IgG seroprevalence between Toowoomba and Redcliffe, when adjusting for the cohort classification and the effect of developmental age since initial cohort sampling. A red dashed line is centered at 0. The density plot quantiles represent the 95% of the density. |

| Sample group | Prevalence in blood spots (positive/tested) | *Bartonella* positives sequenced | Distinct *Bartonella* genotypes | Distinct *Bartonella* clades |
| --- | --- | --- | --- | --- |
| *Age group* |  |  |  |  |
| Juvenile | 79/133 (59%) | 32 | 18 | 2 (A, B) |
| Subadult | 66/84 (79%) | 23 | 11 | 2 (A, B) |
| Adult | 516/619 (83%) | 189 | 47 | 2 (A, B) |
| *SexR* |  |  |  |  |
| Female | 333/425 (78%) | 118 | 38 | 2 (A, B) |
| Male | 328/411 (80%) | 126 | 32 | 2 (A, B) |
| *Site type* |  |  |  |  |
| Continuous | 619/774 (80%) | 207 | 50 | 2 (A, B) |
| Nomadic | 42/64 (66%) | 37 | 16 | 2 (A, B) |
| *Year* |  |  |  |  |
| 2018 | 130/170 (77%) | 70 | 28 | 2 (A, B) |
| 2019 | 331/402 (82%) | 142 | 37 | 2 (A, B) |
| 2020 | 200/266 (75%) | 32 | 13 | 2 (A, B) |

Supp. Table 2. Prevalence of *Bartonella* in bat blood spot FTA cards across demographic variables. Continuously sampled sites included Redcliffe and Toowoomba (both sampled in 2018, 2019, and 2020). Nomadic sites included Gympie (2019), Hervey Bay (2018, 2020), Maclean (2018), and Mount Ommaney (2019).

| 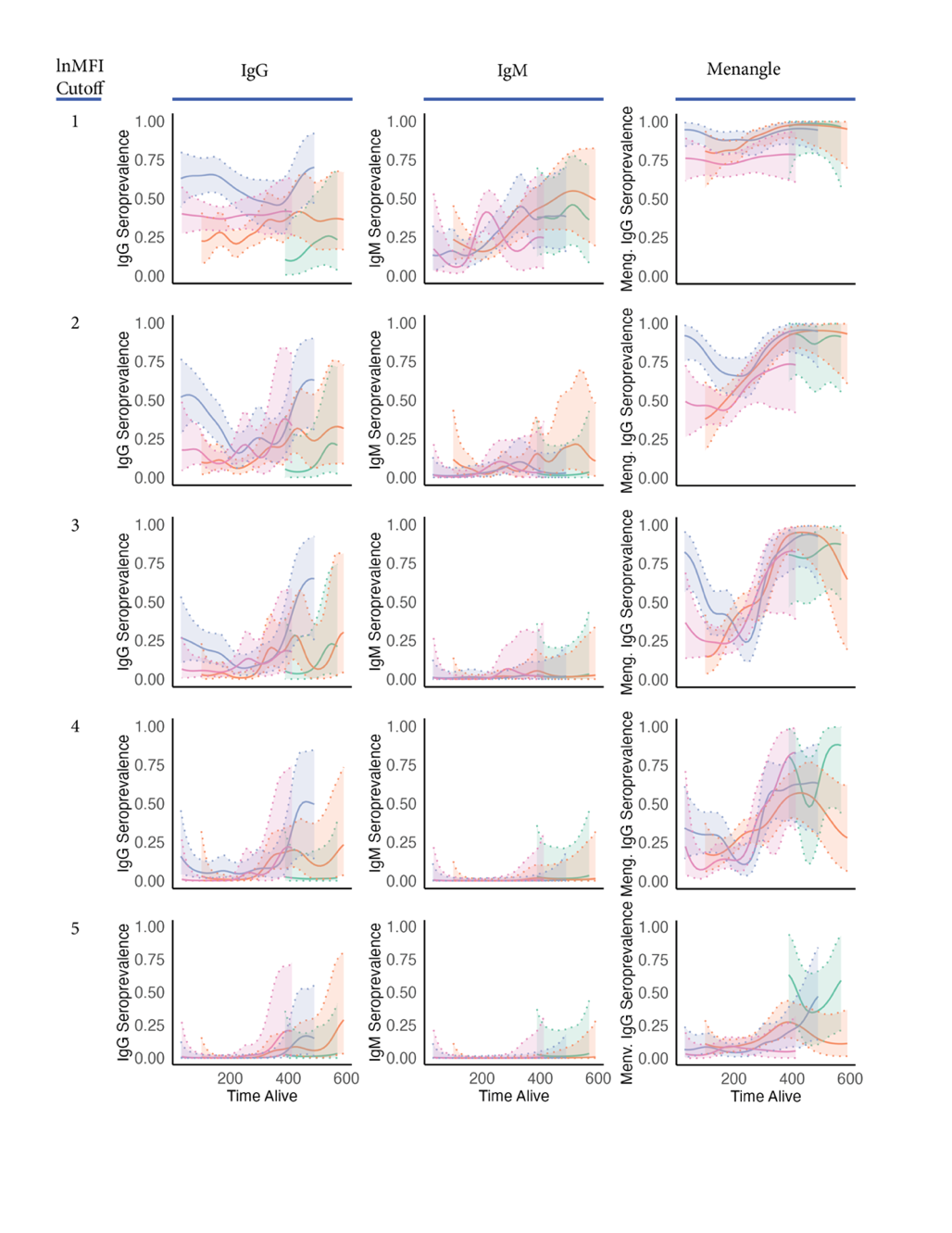 |
| --- |
| Supp. Fig. 15 (A) IgG and IgM dynamics with increasingly stringent integer value cutoffs for lnMFI cutoff to establish seropositivity (1 to 5). The estimates are from our Gaussian smoothing model. The shaded region around the prevalence estimate represents the 95% credible interval from the Gaussian smoothing model. |

Supplemental Methods

*Supplemental Methods Section 1*

For assessing mAb cross-reactivity, we enriched bat serum for specific Ig classes. This was done using size and affinity columns. Serum was passed, separately, once over five affinity columns: A) a nonspecific column CM1 (anti-FeLV), B) CM6E affinity column (anti-IgM), C) IgA5-3b afficol (anti-IgA), D) IgA9-6A afficol (anti-IgA), and E) E6-71 (anti-IgE). We precipitated this effluent serum with ammonium sulfate (NH_4_)_2_SO_4_ at 312 grams/liter to produce a 50% concentrate. The slurry was stirred for 30 minutes at room temperature, spun at 4,000 x G for 35 minutes, and the precipitate was re-dissolved in PBS pH 7.2 up to 12 mL. This 12 mL fraction was divided into two equal fractions, which were run over a 50 cm x 2.5 cm S-300 column and eluted with PBS pH 7.2. Care was taken to run each fraction under identical load conditions. Protein content of the eluted fractions was monitored using a continuous flow spectrophotometer. We collected a fraction every four minutes for 3.5 hours. We tested several monoclonal antibodies (mAbs) for their ability to bind fractions by ELISA (Supp. Fig. 4A) and Western blot (Supp. Fig. 4B) .

To further validate any cross-reactive mAbs, we used standard Western blot procedures. Fractionated serum was heated in 5% β-mercaptoethanol (BME) at 56°C for eight minutes then run on a sodium dodecyl sulfate polyacrylamide gel electrophoresis (SDS-PAGE) followed by transfer to nitrocellulose paper. Primary antibodies CM7 and FDG1-2 were added to the nitrocellulose paper at 1ug/mL for two hours at ambient temperature. Detection was done with secondary anti-mouse HRP-labeled antibodies. All wash steps were done using PBS in 1% Tween-20 in triplicate. A protein standard (Precision Plus Protein™ Kaleidoscope™ Prestained Protein Standards #1610375) was run with every gel.

## *Supplemental Methods Section 2*

*Bartonella* samples with amplicons of the expected size (300 bp) and sufficient DNA concentration following purification were submitted for Sanger sequencing using inner primers. Sequencing was performed using the 3730x1 DNA Analyzer (ThermoFisher, Cat. A41046) at the Cornell Institute of Biotechnology. Forward and reverse reads were assembled using Geneious Prime 2024.0.2 (https://www.geneious.com) with the Geneious Alignment tool. Consensus sequences were constructed using a global alignment with free end gaps and a cost matrix of 65% similarity. Sequences were inspected with the NCBI Basic Alignment Search Tool (BLAST) to confirm amplification of *Bartonella* DNA and to identify closely matching sequences. We added closely matching sequences to a previously assembled database of reference *gltA* sequences from named *Bartonella* species and previous studies of bats and nycteribiid bat flies (Fagre et al., 2023). Sequences were aligned with MAFFT v7 using the local iterative method (L-INS-i) with default parameters (Katoh & Standley, 2013) and then trimmed to equal length using the trimAl tool, removing columns with gaps in more than 20% of the sequences (Capella-Gutiérrez et al., 2009). For any identical sequences, only one representative sequence was retained in the alignment for phylogenetic analysis. A maximum likelihood phylogeny was generated following model selection using IQ-TREE v2 (Minh et al., 2020) and visualized using the GGTREE (GGTREE v3.8.0) package in R (R v4.3.2) (Yu et al., 2017). Groupings of sequences were then assigned into monophyletic clades according to *gltA* sequence identity following previously established guidelines for *Bartonella* (La Scola et al., 2003).

## Supplemental Results

*Supp. Results Section 1: Cohort Model Supplemental Information*

For females, the mean effect estimates of capture date against forearm was 5.9 e-11 (90% Credible Interval (CI) 2.7 e-11, 9.3 e-11) and for weight was 1.3 e-3 (90% CI 1.1 e- 3, 1.5 e- 3). For males, the mean effect estimates of capture date against forearm was 3.7 e-11 (90% CI 4.5 e-12, 8.4 e-11) and for weight was 1.1 e-3 (90% CI 7.7 e- 4, 1.3 e- 3) (Supp. Fig. 2F). The relationship between morphological metrics and developmental age since initial cohort sampling began to diverge between sexes amongst older animals (approximately 100 days and older) (Supp. Fig. 2 D & E).

*Supp. Results Section 2: IgG Seroprevalence Results:*

In our logistic regression analysis, the seropositive percentage was higher in the 2018 birth cohort compared to the 2016, 2017 and 2019 birth cohorts (log odds point estimate and 90% credible interval for 2016: -3.56 {-6.25, -1.67}; 2017: -2.63 {-3.29, -1.98}; 2018: -1.16 {-1.68, -0.609}; and 2019: -2.58 {-4.04, -1.36}; Fig. 2B). We found no linear association (in the log odds space) between the predicted developmental age (days) and the presence of anti-Hendra virus IgG antibodies, while controlling for the effect of cohort (log odds & CI = 0.001 {-0.001, 0.003}; Fig. 2B).

*Supp. Results Section 3: Bartonella Results*

No cohort had higher odds of being Bartonella spp. positive (PE & CI 2017: -0.942 {-2.05, 0.158}; 2018: -0.712 {-1.45, -0.016}; 2019: -0.672 {-1.65, 0.265}) (Fig. 2B). All 2016 cohort samples (n=5) were positive for Bartonella spp., preventing inclusion in the logistic regression due to perfect separation.

We sequenced a subsample (n = 244) of our *Bartonella-*positive samples. We identified 55 distinct genotypes that grouped into two monophyletic clades (Supp. Fig. 10) with high bootstrap support (≥99%). Clade A included 35 sequences grouped into 12 genotypes that shared 93.9–99.7% sequence identity. Sequences in clade A were closely related (93.1–100% identity) to *Bartonella* sequences previously detected in *P. hypomelanus* and associated *Cyclopodia horsfieldi* bat flies in Malaysia as well as in *P. medius* and associated *C. sykesii* bat flies in Bangladesh (Fagre et al., 2023; Hou et al., 2018; Morse et al., 2012). Clade B included 209 sequences among 43 genotypes sharing 95.3–99.7% sequence identity. Clade B sequences were closely related (92.6–100%) to *Bartonella* previously detected in *C. horsfieldi* collected from *P. hypomelanus* in Malaysia, in *P. medius* and *C. skyesii* in Bangladesh, as well as other pteropodid bats and nycteribiid bat flies sampled in Africa and Asia (Bai et al., 2015; Billeter et al., 2012; Brook et al., 2015; Fagre et al., 2023; Kuang et al., 2022; Low et al., 2022; Morse et al., 2012; Wilkinson et al., 2016). Both Clades A and B were consistently detected across all age groups, sexes, site types, and years (Supp. Table 2).

The consistency in the relative frequencies of these two clades (clade B: 86%, clade A: 14% on average) across all demographic variables suggests they share common transmission dynamics. While we could not sequence all positive samples to compare clade-specific dynamics directly, the stable ratio between clades across different cohorts, sexes, age groups, and years indicates that both are transmitted via the same mechanism (nycteribiid bat flies) and that our conclusions about lack of intercohort variation apply to both clades. Future studies with more comprehensive sequencing could examine whether rare Bartonella genotypes show different temporal dynamics, though the stable clade ratios observed here suggest this is unlikely.

*Supp. Results Section 4: IgM Validation:*

We began by identifying and validating anti IgM mAb that could work for *Pteropus* bats. We identified two anti-feline IgM antibodies (CM6E and CM7) that bound S-300 fractions 23-29 and one anti-canine IgG antibody (FDG1-2) that bound S-300 fractions 32 to 34 (Supp. Fig. 4A). In our Western blot analysis (Supp. Fig. 4B), CM7 bound a 75kDA protein in the CM6E affinity column eluate and in the S-300 size fractionated prep tubes 23-29. CM7 did not bind any protein in the S-300 size fractionated tubes 32-34. The FDG1-2 mAb bound a 50kDA protein in the S-300 size fractionated tubes 32-34.

*Supp. Results Section 5: Cutoff stringency, sex, and site have small effects on serological dynamics*

We tested the stringency with which we assigned bats to a cohort. We removed any bats from the dataset for which the model was uncertain about cohort assignment (posterior probability < 95%). Overall, dynamics did not change substantially, except that the 2018 cohort lost anti-Hendra virus IgM antibodies after 300 days of age (Supp. Fig. 13).

Next, we assessed differences by sampling location. For this analysis, we stratified data to our two primary sampling locations (Redcliffe and Toowoomba) and analyzed each separately. There were some qualitative differences in dynamics between Redcliffe and Toowoomba; the 2018 Redcliffe cohort experienced a decline in IgG seroprevalence during the first 200 days since initial cohort sampling, whereas the 2018 Toowoomba cohort maintained moderate seroprevalence throughout their first year (Supp. Fig. 14A). However, including the bat roost location in our model of IgG Hendra virus did not improve model fit (ELPD-LOO-CV estimate ± SE: -182.0 ± 12.7) over the model which included cohort (ELPD-LOO-CV estimate ± SE: -178.8 ± 12.7; ELPD difference ± SE: -3.5 ± 18.1). Similar to IgG, no measurement (IgM, *Bartonella* spp., Hendra virus RNA detection, and Menangle virus IgG detection) had a model fit that was improved by including bat roost sampling location (IgM: ELPD Difference Estimate & SE: -3.4, 16.7; *Bartonella*: ELPD Difference Estimate & SE -2.5, 9.8; PCR Difference Estimate & SE 6.4, 14.0; Menangle IgG Difference Estimate & SE: -5.1, 13.0) (Supp. Fig. 14B).

*Supp. Results Section 6: lnMFI cutoffs do not impact temporal IgG seroprevalence patterns until they become exceedingly stringent*

We were concerned that our lnMFI cutoff, which was fit to all animals in our dataset (adults, subadults, and juveniles), might not capture the serological dynamics in juveniles. Juvenile bats’ B cells have likely not undergone the extensive somatic hypermutation and affinity maturation as adult bats’ B cells, and thus may not have high affinity to our Hendra virus antigen. Furthermore, antibody levels in serum will often wane after initial exposure. Serum levels are more likely to remain detectable, and high, if an animal has been repeatedly exposed, as is the case for adults.

To test this, we assessed a range of lnMFI cutoffs and how these impacted temporal seroprevalence patterns. We used integer cutoffs of lnMFI 1, 2, 3, 4, and 5 (Supp. Fig. 15A) for both IgG and IgM antibodies. We tested Hendra virus G antigen as well as the Menangle virus antigen. As expected, increasing the lnMFI cutoff for IgG and IgM resulted in a reduction in the number of bats classified as seropositive. However, there were surprising findings, especially when broken down by antibody class. First, IgM seroprevalence curves were especially sensitive to increasing the cutoff. This is not surprising, as mammalian IgM antibodies have lower affinity and are less specific that IgG. What was surprising, was the IgG sensitivity. Overall, while many of our IgG findings remained consistent (notably, the 2018 cohort remained higher than the other cohorts), maternal antibodies for all cohorts were especially sensitive to higher cutoffs. We observed a similar pattern when we looked at the Menangle virus antibodies, indicating this is not a Hendra virus–specific phenomenon.

Supp. Results Section 7:

We compared the 2018 and 2019 cohorts alone. We did this supplementary analysis because these were the two cohorts where we had the most data collected on the youngest juvenile bats. When we did this analysis, we again found that including a cohort effect improved the model for IgG (ELPD difference: -5.0 ± 15.0 SE for the no-cohort model), but not IgM (ELPD difference: -3.0 ± 16.1 SE), *Bartonella* (ELPD difference: -1.1 ± 7.2 SE), or HeV PCR detection. Menangle virus IgG levels also showed improved fit with cohort effects included (ELPD difference: -3.0 ± 10.1 SE for the no-cohort model). The standard errors for all comparisons exceeded the ELPD differences, indicating uncertainty in model selection, though the IgG model showed the strongest support for including cohort effects.
