# Supplementary material for: Cohorts of immature Pteropus bats show interannual variation in Hendra virus serology
Source: J Anim Ecol. 2026 Feb 1;95(3):521–37. doi: 10.1111/1365-2656.70213 (PMC12957713; doi:10.1111/1365-2656.70213)
Supplement: Supplementary file 2 — Table S1. Prevalence of Bartonella in bat blood spot FTA cards across demographic variables. Continuously sampled sites included Redcliffe and Toowoomba (both sampled in 2018, 2019 and 2020). Nomadic sites included Gympie (2019), Hervey Bay (2018 and 2020), Maclean (2018) and Mount Ommaney (2019). [file JANE-95-521-s002.docx]

| Sample group | Prevalence in blood spots (positive/tested) | *Bartonella* positives sequenced | Distinct *Bartonella* genotypes | Distinct *Bartonella* clades |
| --- | --- | --- | --- | --- |
| *Age group* |  |  |  |  |
| Juvenile | 79/133 (59%) | 32 | 18 | 2 (A, B) |
| Subadult | 66/84 (79%) | 23 | 11 | 2 (A, B) |
| Adult | 516/619 (83%) | 189 | 47 | 2 (A, B) |
| *SexR* |  |  |  |  |
| Female | 333/425 (78%) | 118 | 38 | 2 (A, B) |
| Male | 328/411 (80%) | 126 | 32 | 2 (A, B) |
| *Site type* |  |  |  |  |
| Continuous | 619/774 (80%) | 207 | 50 | 2 (A, B) |
| Nomadic | 42/64 (66%) | 37 | 16 | 2 (A, B) |
| *Year* |  |  |  |  |
| 2018 | 130/170 (77%) | 70 | 28 | 2 (A, B) |
| 2019 | 331/402 (82%) | 142 | 37 | 2 (A, B) |
| 2020 | 200/266 (75%) | 32 | 13 | 2 (A, B) |

Supp. Table 1. Prevalence of *Bartonella* in bat blood spot FTA cards across demographic variables. Continuously sampled sites included Redcliffe and Toowoomba (both sampled in 2018, 2019, and 2020). Nomadic sites included Gympie (2019), Hervey Bay (2018, 2020), Maclean (2018), and Mount Ommaney (2019).
